# Burden of Chikungunya Fever and Its Economic and Social Impacts Worldwide: A Systematic Review

**DOI:** 10.1111/tmi.70012

**Published:** 2025-07-22

**Authors:** Vaneide Daciane Pedí, Giovanny Vinícius Araújo de França, Viviane Bellini Rodrigues, Felipe Tavares Duailibe, Marcella T. P. Santos, Maria Regina Fernandes de Oliveira

**Affiliations:** ^1^ Fundação Oswaldo Cruz Brasília Federal District Brazil; ^2^ Faculdade de Medicina, Programa de Pós‐Graduação em Medicina Tropical Universidade de Brasília Brasília Federal District Brazil; ^3^ Ministério da Saúde do Brasil, Secretaria de Ciência, Tecnologia e Inovação e do Complexo Econômico Industrial da Saúde, Departamento de Ciência de Tecnologia Brasília Federal District Brazil; ^4^ Departamento de Nutrição Universidade de Brasília Brasília Federal District Brazil; ^5^ British Columbia Centre for Excellence in HIV/AIDS Vancouver British Columbia Canada; ^6^ Centro Universitário de Brasília Brasília Federal District Brazil

**Keywords:** burden of disease, Chikungunya, disability‐adjusted life years, disease costs, economic and social impact, economic studies, quality of life, systematic review

## Abstract

**Objectives:**

This study aimed to investigate the social and economic impacts and disease burden of Chikungunya Fever globally through a systematic literature review.

**Methods:**

We performed a comprehensive literature search through MEDLINE (via PubMed), LILACS, and Embase databases, and grey literature, including studies of populations diagnosed with Chikungunya Fever or at risk of infection published in English, Spanish, French, or Portuguese, without date restrictions. Two reviewers independently performed study selection, data extraction, and quality assessment. Methodological quality was assessed using different tools.

**Results:**

Forty‐three publications were included. Until 2013, publications originated solely from the Asian and African continents. From 2015 onwards, South America emerged as the predominant source. Publications were classified as cost studies (25), including cost‐of‐illness (18) and program cost (6); burden of disease studies (10); cost‐outcome studies (4), including cost‐effectiveness (3) and cost‐utility (1); and quality‐of‐life studies (15). Reported total direct costs associated with Chikungunya Fever ranged from US$ 3.5 million (US Virgin Islands, 2014–2015) to US$ 83.6 billion (Region of the Americas, 2013–2015). Direct medical costs varied from US$ 308.94 (Tamil Nadu, India, 2006) to US$ 33.7 million (Réunion Island, 2005–2006). Vector control program costs ranged from US$ 888,000 annually (Greece, 2013–2017) to US$ 466 million (Brazil, 2016). Estimated disability‐adjusted life years per 100,000 population ranged from 4.53 (India, 2006) to 2432 (Region of the Americas, 2013–2015). Quality‐of‐life studies demonstrated substantial declines across multiple domains, indicating significant functional impairment due to Chikungunya Fever.

**Conclusion:**

Chikungunya Fever imposes a considerable economic and social burden, surpassing that of other endemic arboviral diseases such as dengue and yellow fever. These findings underscore the need for further research to accurately quantify the full scope of Chikungunya Fever‐related costs and impacts on affected populations.

## 1 Introduction

Chikungunya fever (CHIKF) is an arboviral disease caused by the Chikungunya virus (CHIKV), an arthritogenic Alphavirus that belongs to the Togaviridae family [1]. The CHIKV is primarily transmitted to humans by infected female mosquitoes of the *Aedes* genus. *Aedes aegypti* is reported as the primary vector in urban outbreaks and epidemics, operating through the mosquito–human–mosquito transmission cycle [2, 3]. The first laboratory‐confirmed CHIKF epidemic in humans occurred in Tanzania from 1952 to 1953 [4, 5].

From the 2000s to the 2010s, a shift in the status of CHIKF was observed, transitioning from a self‐limiting, mild acute health condition confined to African and Asian countries to one of the primary arboviral diseases with global impact [6, 7]. Due to its explosive nature, with a high potential for causing large‐scale outbreaks and epidemics [8], combined with the widespread distribution and adaptation of its vector across all continents, CHIKF has emerged as a significant international public health concern. In a globalised context, infected travellers play a critical role in introducing the virus to previously unaffected areas. Additionally, mutations in the East/Central/South African (ECSA) genotype [9] have markedly enhanced the transmission efficiency of the virus [10].

Since the 2010s, CHIKF outbreaks have been reported across nearly all continents, including Africa, the Americas, Asia, Europe and islands in the Indian and Pacific Oceans. Globally, between 2010 and 2023, more than 10 million cases of CHIKF were reported across the Americas, Africa, Asia, Europe, and the Indian and Pacific Oceans [11, 12]. CHIKV reemerged in the Americas in 2013, with widespread transmission occurring in 2014 and 2015 [13]. A decade after its reintroduction, the disease resurfaced, and between epidemiological weeks (EW) 01 and 52 of 2023, 410,754 cases and 419 deaths were reported from 17 countries and territories in the region, marking the highest number of reported cases in recent years [14].

Despite its low case fatality rate (0.5 to 1.3 per 1000 cases), the expansion of the disease, together with the concurrent circulation of other arboviral diseases such as dengue, Zika and Oropouche, raises concerns regarding issues such as underreporting of cases and deaths [15], as well as the increasing severity of the disease in the acute, post‐acute and chronic phases [16]. In this context, clinical manifestations previously considered rare are now being observed more frequently: 25%–50% of patients develop long‐term disabilities that significantly impair their quality of life (QoL) [17, 18], including severe atypical cases and even death, particularly among the elderly and children under 1 year of age.

Outbreaks, epidemics or sustained transmission impose significant economic and social burdens on individual and collective health and healthcare systems and services [14]. Despite this, CHIKF remains largely neglected [8], and studies on the economic and social impacts of the disease are scarce. The economic impacts of a disease comprise direct, indirect and intangible costs. Direct costs are those associated with healthcare expenditures and can be further classified into healthcare (medical) and non‐healthcare (non‐medical) costs. Indirect costs are related to productivity losses, absenteeism or premature death. In contrast, intangible costs refer to losses in QoL due to pain, suffering or social exclusion resulting from the disease [19, 20].

This study aimed to investigate the social and economic impacts and disease burden of sustained transmission, outbreaks and epidemics of CHIKF worldwide through a systematic literature review.

## 2 Materials and Methods

### 2.1 Study Design and Registration Protocol

This study is a systematic literature review, with its protocol registered in the International Prospective Register of Systematic Reviews (PROSPERO) under the registration number CRD42022367956. A descriptive approach was employed following the Preferred Reporting Items for Systematic Reviews and Meta‐Analyses (PRISMA) guidelines [21]. Ethics approval was not required for this study, as it was based exclusively on the analysis of publicly available published data.

### 2.2 Research Question

The study was guided by the following research question: ‘What is the social and economic impact, as well as the impact in disability‐adjusted life years (DALYs) and quality‐adjusted life years (QALYs), of sustained transmission, outbreaks, and epidemics of CHIKF worldwide?’ Table 1S details the search strategy framework, which follows the PICOT acronym (Population, Intervention, Comparison, Outcome and Type of study design).

### 2.3 Eligibility Criteria

We included primary studies on health economic evaluation, cost‐of‐illness analyses, program cost assessments, studies assessing the burden of the disease, and qualitative investigations focused on social and economic losses. Eligible studies had to report at least one of the following outcome measures:

- Disability‐Adjusted Life Years (DALYs): expresses years of life lost to premature death and years lived with a disability of specified severity and duration. It is calculated as follows: DALY = YLL + YLD [22].
- Years of life lost due to premature mortality (YLLs): quantifies the years of potential life lost due to premature death. It is calculated by multiplying the number of deaths at each age by the standard life expectancy remaining at that age [22].
- Years lived with disability (YLDs): quantifies the impact of non‐fatal health outcomes by estimating the years lived with reduced health status as a result of disease or injury. It is calculated by multiplying the prevalence of a condition by a disability weight, which reflects the severity of the health loss, and by the average duration of the condition [22].
- Quality‐adjusted life‐year (QALYs): quantify the total amount of quality‐adjusted health an individual experiences over a given period. One QALY represents 1 year lived in perfect health or in a state perceived by the individual as equivalent to full health [23].
- Direct costs: refer to expenditures associated with patient care and can be categorised into two components: direct medical costs and direct non‐medical costs [24].
- Direct medical costs: include costs directly attributable to the primary disease, as well as those associated with comorbid conditions arising from the disease itself or its treatment, such as consultations, hospitalisations, medications and diagnostic procedures including specific laboratory tests [24].
- Direct non‐medical costs: refer to expenses arising from illness that are not directly tied to the provision of medical services or treatments, including accommodation, caregiver expenses and related expenditures [24].
- Indirect costs: represent costs related to productivity loss due to illness, including lost income from work absenteeism [24].
- Costs related to vector prevention and control: include all expenditures aimed at reducing or interrupting the transmission of vector‐borne diseases.
- Catastrophic health expenditures: occur when out‐of‐pocket health expenditures surpass a defined proportion of household income and its capacity to pay, leading to financial hardship or risk of impoverishment [25].

Inclusion and exclusion criteria for study selection were defined a priori and applied systematically. Eligible studies comprised national and international publications, indexed from the inception of each database through December 31, 2023, and published in English, Spanish, French or Portuguese. Review articles, opinion pieces, short communications and experimental studies were excluded from the analysis.

### 2.4 Search Terms and Strategies

Search terms were identified through iterative pilot searches, which informed the development of search syntaxes incorporating terms and keywords that maximised search sensitivity (Table 2S). The original search strategy was developed for the MEDLINE database (via PubMed) and subsequently adapted for use in the following databases: Embase, accessed through the Coordination for the Improvement of Higher Education Personnel—CAPES/MEC Portal; and LILACS, accessed via the Virtual Health Library (*Biblioteca Virtual em Saúde*, in Portuguese). The complete search strategies for each database are provided in Tables 3S–5S.

### 2.5 Selection and Screening of Publications

Systematic searches across the selected databases were initially conducted on December 31, 2022, and updated in March 2024. Metadata from the retrieved publications were exported to Mendeley (https://www.mendeley.com), which was also used to identify and remove duplicate records. Following de‐duplication, two independent reviewers (V.D.P. and V.B.R.) screened the titles and abstracts of the publications using Rayyan (http://rayyan.qcri.org), a web‐based application designed to support systematic reviews. The reviewers independently selected studies that aligned with the scope of the research question based on the relevance of their reported data.

Two reviewers (V.D.P. and V.B.R.) independently conducted full‐text screening to determine which studies met the inclusion criteria for this systematic review. Reviewer discrepancies were resolved through discussion and consensus at both stages of the selection process. In cases where disagreement persisted, a third reviewer (M.R.F.d.O.) was consulted to provide arbitration and make the final decision regarding study inclusion.

A complementary search was also conducted to identify relevant publications within the grey literature. This search included thesis and dissertation databases from CAPES/MEC, the University of Brasília (UnB) and the University of São Paulo (USP), using the keyword ‘Chikungunya’. No filters related to study type were applied during this search. Two independent reviewers (V.D.P. and V.B.R.) also screened and selected grey literature.

Additionally, the reference lists of key systematic reviews and meta‐analyses were screened to ensure that all relevant studies had been captured by the initial database searches.

### 2.6 Data Extraction and Analysis

Metadata from the included studies were recorded in duplicate using a pre‐defined data extraction framework. Data synthesis was conducted following the Methodological Guidelines for Economic Evaluations published by the Brazilian Ministry of Health [26]. The following variables were extracted from each study: study location, geographic scope, healthcare setting (public/private), study period, type of study and evaluation, target population and its characteristics, sample size, analytical perspective, time horizon, cost data, YLL, YLD, disability weights, DALYs, QALYs and the type of QoL instrument used. Studies that assessed multiple outcomes were classified into all relevant analytical categories, where appropriate.

To facilitate cost comparisons across different currencies, all monetary values were converted to US dollars (USD) based on the exchange rate as of July 1st of the respective year of data collection. For studies covering multiple years, values were converted based on the midpoint year of the study period.

The cost estimation methodology proposed by Silva et al. [20] was adopted to analyse and classify healthcare costs, following six key steps that should be considered: (i) definition of the study perspective (who bears the costs associated with the technology or strategy under investigation?); (ii) determination of the time horizon (for how long will the costs be estimated?); (iii) identification of costs (which cost items will be included in the analysis?); (iv) measurement of costs (what is the unit of measurement for each cost item?); (v) selection of the method for cost valuation (how will values be assigned to the cost units?) and (vi) temporal adjustments (is the time horizon longer than 1 year?).

### 2.7 Quality Assessment

We applied the following tools to assess the methodological quality of the selected publications: ‘Economic Evaluation of Health Technologies: A Critical Review Framework’ [27]; ‘Checklist for Analytical Cross‐Sectional Studies – Critical Appraisal Tools for Use in JBI Systematic Reviews’ [28] and ‘A Consensus‐Based Checklist for the Critical Appraisal of Cost‐of‐Illness (COI) Studies’ [29].

Studies were categorised based on quality score ranges adapted from the works of Rodrigues et al. [30] and Psaltikidis et al. [31], as follows: high (80%–100%), medium‐high (60%–79%), medium‐low (40%–59%) or low (< 40%). These checklists outline the minimum set of information that should be provided in each category when reporting evaluation studies, assisting in assessing the publications' reliability, relevance and key findings [32].

## 3 Results

### 3.1 Study Selection

**FIGURE 1:**
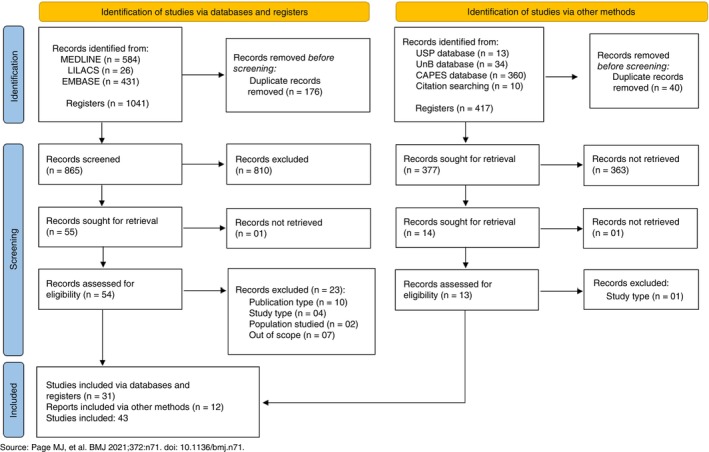
Flowchart of the selection process for the systematic review of the economic and social impact of Chikungunya Fever.

A total of 1041 records were identified across the three databases searched. After removing duplicate publications (*n* = 176), 865 records were screened based on title and abstract to assess their eligibility according to the inclusion criteria. We could not obtain the full text of one of the 55 selected records. The full text of the remaining 54 publications was reviewed, and 31 articles were included in this review. Twenty‐three publications were excluded because they did not meet the eligibility criteria regarding the publication type (*n* = 10), study design (*n* = 04) and population studied (*n* = 02), and seven of them were out of scope (Figure 1).

The screening of the reference lists of key systematic reviews and meta‐analyses, and the search of the grey literature, through thesis and dissertation databases, yielded 417 records related to the search term ‘chikungunya’. Of these, 360 were retrieved from the CAPES/MEC portal, 34 from the University of Brasília (UnB) thesis and dissertation database, 13 from the University of São Paulo (USP) database, and 10 through citation searching. After removing 40 duplicate publications, 377 were screened based on their title and abstract, of which 363 were excluded for not meeting the eligibility criteria, and one publication was excluded because it was not publicly available. Thirteen publications were reviewed in full, of which 12 were included in this review and one was excluded for not meeting the inclusion criteria (Figure 1).

### 3.2 Characteristics of the Selected Studies

**FIGURE 2:**
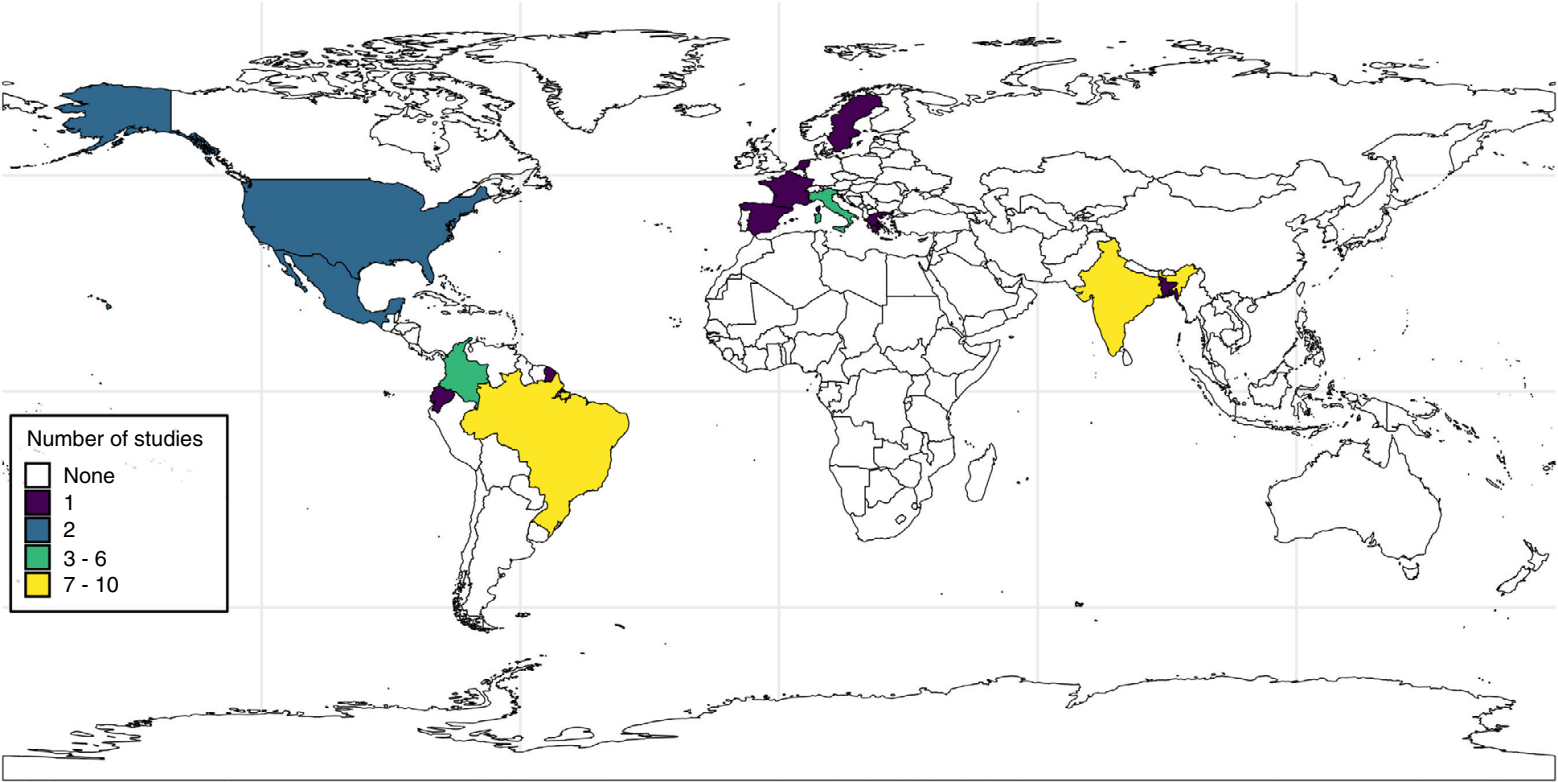
Number of retrieved studies by country, 2009–2023. *Note*: The number of studies was categorised using the Jenks natural breaks classification method to optimise the grouping based on data distribution.

Forty‐three publications were included in this systematic review, spanning 14 years from 2009 to 2023 (Table 6S). Most studies (74.4%) were published from 2017 onwards. Until 2013, all published studies originated exclusively from the Asian and African continents, accounting for 67% and 33%, respectively. From 2015 onwards, South America emerged as the continent with the highest concentration of publications. The geographical distribution of studies was determined based on the origin of the data analysed. Most published studies originated from South America (58.8%), followed by Asia (26.5%) and Europe (14.7%). Regarding the country of origin, Brazil (10 studies), India [8], and Colombia [6] accounted for 58.8% of the publications included (Figure 2). Approximately 67% of the studies used data from the public health sector as their primary source, while 30.2% adopted a mixed (public and private) approach. Most analyses were conducted using data at the local or national level, with 15 studies (34.9%) in each category (Table 1).

**TABLE 1:** Distribution of studies included in the systematic review according to the geographical origin of the data studied, type of evaluation, and other characteristics (*n* = 43).

Features	*n*	%
**Publication year**		
2009	3	7.0
2010	1	2.3
2011	1	2.3
2012	2	4.7
2013	1	2.3
2014	0	0.0
2015	2	4.7
2016	1	2.3
2017	6	14.0
2018	5	11.6
2019	6	14.0
2020	3	7.0
2021	3	7.0
2022	8	18.6
2023	1	2.3
**Continent of origin**		
Africa	3	8.8
Americas	1	2.9
Asia	9	26.5
Europe	5	14.7
Europe and North America	1	2.9
North America	4	11.8
South America	20	58.8
**Country**		
Bangladesh	1	2.3
Brazil	10	23.3
Colombia	6	14.0
Curaçao/Kingdom of the Netherlands	3	7.0
Ecuador	1	2.3
France	1	2.3
Greece	1	2.3
Guadeloupe Island/French Department	1	2.3
India	8	18.6
Italy	3	7.0
Mexico	2	4.7
Netherlands, Sweden, Italy, Spain and USA	1	2.3
Reunion Islands/French Department	3	7.0
United States of America	1	2.3
Not applicable (Region of Americas)	1	2.3
**World Bank Classification**		
Lower middle‐income	9	20.9
Upper middle‐income	19	44.2
High‐income	14	32.6
Not applicable (Region of Americas)	1	2.3
**Classification based on the analytical approach**		
Cost studies		
Cost‐of‐Illness^a^	18	52.9
Program cost^a^	6	17.6
Burden‐of‐disease studies	10	29.4
Cost‐outcome studies		
Cost‐effectiveness	3	8.8
Cost‐utility	1	2.9
Quality‐of‐Life studies^a^	15	44.1
**Scenario**		
Private	1	2.3
Public	29	67.4
Public and private	13	30.2
**Coverage**		
Local	15	34.9
Municipal	5	11.6
State	6	14.0
National	15	34.9
Regional	1	2.3
Continental	1	2.3
**Quality Assessment**		
Cost studies		
Medium‐high	13	52.0
High	12	48.0
Cost‐outcome studies		
Medium‐high	2	50.0
High	2	50.0
Quality‐of‐Life studies		
Medium‐high	0	0.0
High	14	100.0

*Note*: The World Bank divides economies into income groups according to 2023 gross national income (GNI) per capita, calculated using the World Bank Atlas method.

^a^ Include studies with a mixed approach (more than one component analysed in the same study).

Twenty‐four cost studies were included, of which 18 [33, 34, 35, 36, 37, 38, 39, 40, 41, 42, 43, 44, 45, 46, 47, 48, 49, 50] assessed the cost of CHIKF, and 6 [41, 46, 51, 52, 53, 54] reported programme costs. Among the cost‐of‐illness studies, 14 [33, 34, 35, 36, 37, 38, 39, 40, 41, 43, 44, 45, 50] estimated direct medical and non‐medical costs, while 16 reported on indirect costs [33, 34, 35, 36, 37, 38, 39, 41, 43, 44, 45, 46, 48, 49, 50, 54]. In addition, 10 studies assessed the CHIKF burden through DALYs [34, 35, 37, 38, 42, 44, 46, 55, 56, 57]. We also report on four cost‐outcome [58, 59, 60, 61] and 15 QoL studies [40, 62, 63, 64, 65, 66, 67, 68, 69, 70, 71, 72, 73, 74, 75] (Table 2).

**TABLE 2:** Main characteristics of cost‐of‐illness, program cost, and burden‐of‐disease studies.

Author (year of publication)	Year(s) studied	Location (sample)	Type of study	Direct costs		Indirect costs	Burden‐of‐disease (DALY/YLD)	Out‐of‐pocket expenses	Program cost
				Medical	Non‐medical	Productivity loss			
Alvis‐Zakzuk et al. 2018 [33]	2014	Colombia (126 patients; 67 children and 59 adults)	Cost‐of‐Illness	Median cost in children: US$ 257.9 Median cost in adults: US$ 66.6	—	Median expenditures: US$ 81.3 per adult patient	—	Median expenditures US$ 5.0 US$ 0.8 for transport US$ 4.2 for drugs	—
Bloch 2016 [34]	2013–2015	America (39.9 million CHIKV cases)	Cost‐of‐Illness Burden‐of‐disease	*Direct costs* US$ 83.6 billion		US$ 101.4 billion (54.9% of total costs)	23.8 million DALYs 2432 DALYs per 100,000 population	—	—
Canali et al. 2017 [52]	2008–2011	Italy (280 municipalities with 4.2 million inhabitants)	Program cost	—	—	—	—	—	Mean values per inhabitant 2008: €1.88 [US$ 2.97] 2009: €1.51 [US$ 2.13] 2010: €1.33 [US$ 1.63] 2011: €1.21 [US$ 1.75]
Cardona‐Ospina et al. 2015 [35]	2014	Colombia (106,592 cases)	Cost‐of‐Illness Burden‐of‐disease	Acute and post‐acute phase: US$ 76.61 Chronic phase: US$1362.13 to US$3319.96	—	US$ 60.2 per patient	40.44 to 45.14 DALYs per 100,000 population (96% during the chronic phase)	—	—
de Margarette et al. 2022 [36]	2017	Fortaleza, Brazil (2683 patients)	Cost‐of‐Illness	*Overall* US$ 383,514.40 *Emergencies* US$ 174,322.91 (45.5%) *Hospitalizations* US$ 194,700.59 (50.8%)	—	US$ 14,490.90 (Absenteeism from work)	—	—	—
Feldstein et al. 2019 [37]	2014–2015	U.S. Virgin Islands (86–165 laboratory‐positive CHIKV cases)	Cost‐of‐Illness Burden‐of‐disease	*Total direct cost*: US$ 3.5 million Acute phase: US$ 2.9 million Post‐acute phase: US$ 0.6 million (19%)		US$ 29.7 million (Cost of absenteeism for acute and long‐term CHIKV illness up to 12 months)	599 to 1322 YLDs associated with long‐term sequelae	—	—
Gonçalves 2021 [38]	2019	Municipality of Rio de Janeiro, Brazil (37,904 notified CHIKF cases)	Cost‐of‐Illness Burden‐of‐disease	Total direct cost: R$ 24,152,966.45 [US$ 6,310,856.14] Acute phase: R$ 10,967,229.00 [US$ 2,865,594.36] Post‐acute phase: R$ 6,329,394.00 [US$ 1,653,788.37] Chronic phase: R$ 6,587,728.00 [US$ 1,721,287.69] Hospitalizations: R$ 84,229.50 [US$ 22,008.07] Diagnosis: R$ 184,385.95 [US$ 48,177.65]		Total indirect cost: R$ 103,220,338.92 [US$ 26,970,132.70] Acute phase: R$ 9,869,248.93 [US$ 2,578,706.45] Post‐acute and chronic phases: R$ 93,351,090.00 [US$ 24,391,426.25]	6.977 DALY 110.40 DALY per 100,000 population	—	—
Gopalan and Das 2009 [39]	2007	Orissa, India (150 respondents)	Cost‐of‐Illness	Median in US$: Diagnosis: 32.1 Drugs and consultation: 30	Median in US$: Transport: 7.1 Stay and food: 6.8 Escort: 7.1	Loss of income US$ 75 (35 median work days lost)	—	Median out‐of‐pocket health care expenditure US$ 84	—
Heydari et al. 2017 [51]	2015	Machala, Ecuador (40 households in a high‐risk community)	Program cost	—	—	—	—	Cost related to the acquisition of mosquito control products is equivalent to 1.90% of weekly average income of the households surveyed	Median household expenses related to the acquisition of mosquito control products: US$2.00 per week (US$ 0 to 9.21)
Hossain et al. 2018 [40]	2017	Dhaka city, Bangladesh (1326 CHIKF cases)	Cost‐of‐Illness	Overall treatment cost: Confirmed cases: US$ 99.3 Probable cases: US$ 26		—	—	—	—
Kaur et al. 2022 [50]	2018–2019	Ahmedabad and Kheda district of Gujarat, India (60 CHIKF cases)	Cost‐of‐Illness	Total Direct Medical cost *(Mean in INR)*: Rs 5347 [US$ 77.56] Consultancy Cost: Rs 368 [US$ 5.34] Diagnosis Cost: Rs 1307 [US$ 18.96] Medicine cost: Rs 4403 [US$ 63.87] Hospitalisation cost (private hospital): Rs 7667 [US$ 111.22]	Total Nonmedical Cost *(Mean in INR)*: Rs 1865 [US$ 27.05] Transportation cost: Rs 569 [US$ 8.25] Special food cost: Rs 945 [US$ 13.71] General food cost while staying: Rs 335 [US$ 4.86]	Total indirect cost *(Mean in INR)*: Rs 4982 [US$ 72.27] (patient and caretaker) Average absenteeism: 15 days Average absenteeism of family members due to illness: 7 days	—	13.3% (8/60) of families use saving during illness 23.3% (14/60) of families had to borrow money for treatment	—
Kolimenakis et al. 2019 [41]	2013–2017	Greece (Imported cases)	Cost‐of‐Illness Program cost	Hospitalisation cost: €2691 [US$ 2987.01] (13 travellers)	—	Productivity losses (during hospitalisation): €818 [US$ 907.98]	—	—	Cost of the current mosquito control program: €800,000 to €1,330,000 per year [US$ 888,000 to 1,476,300]
Krishnamoorthy 2009 [42]	2006	India (National)	Cost‐of‐Illness Burden‐of‐disease	—	—	(INR) Rs. 391 million [US$ 8,506,596]	25,588 DALYs 45.26 DALYs per million population (4.526 per 100,000 population)	—	—
Man et al. 2022 [55]	2014–2019	Rio de Janeiro, Brazil (42,636 confirmed CHIKF cases)	Burden‐of‐disease	—	—		Average yearly DALYs Clinical: 3389 Average Yearly DALYs Lab: 2636	—	—
Mora‐Salamanca et al. 2020 [56]	2013–2016	Colombia (National)	Burden‐of‐disease	—	—		DALYs due to arboviruses: 491,629.2 DALYs due to CHIKF: 350,531.62 (71.3% of the total)	—	—
Nandha and Krishnamoorthy 2009 [43]	2006	Tamil Nadu, India (89 houses surveyed)	Cost‐of‐Illness	Total cost incurred (in INR): Rs 14,200 [US$ 308.94]	Total cost incurred (in INR): Rs 1770 [US$ 38.51] Travel: Rs 18.10 [US$ 0.39] per person Escort: Rs 24.05 [US$ 0.52] per person	Total income loss (in INR): Rs 338,400 [US$ 7362.23] Rs 543 [US$ 11.81] per person	—	—	—
Salinas‐López et al. 2018 [53]	2016	Municipalities of Girón and Guadalajara de Buga, Colombia	Program cost	—	—	—	—	—	Girón US$ 146,651 (US$ 0.88 per capita) Guadalajara de Buga US$ 97,936 (US$ 0.99 per capita)
Seyler et al. 2010 [44]	2006	Mallela village, Kadapa district, Andhra Pradesh state, India	Cost‐of‐Illness Burden‐of‐disease	Mallela: US$ 7800 (US$32 per patient)	—	Mallela: US$ 2200 (US$ 8.9 per case)	Mallela: 6.6 DALYs (average: 0.027 DALYs) Kadapa: 120 to 80 DALYs Andhra Pradesh: 4900 to 7400 DALYS	Mean value for Mallela village: US$ 25.20	—
Soumahoro et al. 2011 [45]	2005–2006	Réunion Island, France	Cost‐of‐Illness	€26.5 million [US$ 33,689,450.00] €90 for each outpatient [US$ 114.42] €2000 for each inpatient [US$ 2542.60]	—	Productivity costs: €17.4 million [US$ 22,120,620.00]	—	—	—
Teich et al. 2017 [46]	2016	Brazil (National)	Cost‐of‐Illness Burden‐of‐disease Program cost	—	—	Arboviruses: R$ 431 million [US$ 133,916,872.00] CHIKF: R$ 123,943,728.00 [US$ 38,510,803.61] 29% of the total	0.036 DALYs per episode of disease	—	Vector combat: R$ 1.5 billion [US$ 466,068,000] Acquisition of insecticides and larvicides: R$ 78.6 million [US$ 24,421,963]
Tozan et al. 2023 [47]	2016–2020	Amsterdam (The Netherlands); Stockholm (Sweden); Brescia and Negrar (Italy); Madrid and Barcelona (Spain); Cambridge and New York City (USA)	Cost‐of‐Illness	—	—	Lost income: US$ 2400 (2 patients)	—	Hospitalised abroad: US$ 108 (1 patient) Ambulatory abroad: US$ 120 (1 patient)	—
Vázquez‐Cruz et al. 2018 [48]	2015	Guerrero, Mexico (12,062 patients)	Cost‐of‐Illness	—	—	MXN 2,397,393.40 pesos [US$ 152,505.36]	—	—	—
Vazquez‐Prokopec et al. 2022 [54]	2018–2019	Yucatan, Mexico (3780 houses)	Program cost	—	—	—	—	—	US$ 4.2 to US$ 10.5 per house treated
Vidal et al. 2022 [57]	2016–2017	Brazil (National)	Burden‐of‐disease	—	—	—	2016: 77,422.61 DALYs (0.3757 per 1000 inhab.) 2017: 59,307.59 DALY (0.2856 per 1000 inhab.)	—	—
Vijayakumar et al. 2013 [49]	2007	Kerala, India (3623 people from 857 households)	Cost‐of‐Illness	Mean out‐of‐pocket expenditure: US$ 11.7 (Doctor's fees, medicine and investigation charges)	Mean out‐of‐pocket expenditure: US$ 4.1 (transportation, food and others)	Mean economic loss due to work days lost US$ 29.4	—	Mean out‐of‐pocket expenditure: US$ 15.6	—

*Note*: All monetary values were converted to US dollars (USD) for the year in which the data were collected. The original values are presented followed by their corresponding USD equivalents in square brackets.

In addition, 8 (18.6%) of the 43 reviewed studies estimated the economic impact of CHIKF at the family level and patients' out‐of‐pocket expenses. The costs encompassed expenditures related to medical care, diagnosis, follow‐up during CHIKF treatment, resources spent on vector control, and income loss due to absenteeism. Six studies [33, 40, 44, 50, 51, 54] focused on out‐of‐pocket expenses, while two [39, 49] estimated catastrophic costs. These studies will be presented following the classification based on the analytical approach, as shown in Table 1.

### 3.3 Cost‐of‐Illness Studies

#### 3.3.1 Direct Medical and Non‐Medical Costs

Direct costs associated with patient care were the primary cost components estimated in 14 studies, including medical costs, such as medical consultations, hospitalisations, laboratory and imaging tests, and pharmaceuticals, and non‐medical costs, such as transportation, accommodation and expenses for accompanying persons (e.g., food, transport, and lodging) (Table 3). Three studies [34, 37, 38] reported the total direct cost related to CHIKF, which ranged from US$ 3.5 million in the US Virgin Islands (2014–2015) [37] to US$ 83.6 billion in the Region of the Americas (2013–2015) [34]. The total medical direct costs varied from US$ 308.94 in Tamil Nadu, India in 2006 (89 houses surveyed) [43] to US$ 33.7 million in Réunion Island (2005–2006) [45].

**TABLE 3:** Distribution of the main estimated cost items reported in the studies included in the review.

Item	No. of articles	References
Laboratory and imaging tests	12	[33, 36, 38, 39, 40, 41, 43, 45, 46, 47, 49, 50]
Medical consultations	11	[33, 36, 38, 39, 40, 43, 45, 46, 47, 49, 50]
Medication	11	[33, 36, 38, 39, 40, 43, 45, 46, 47, 49, 50]
Food	8	[36, 39, 40, 43, 46, 49, 50, 54]
Transportation	8	[39, 40, 43, 46, 49, 50, 53, 54]
Hospitalisation	6	[33, 36, 39, 45, 49, 50]
Escort (food, transportation and hotel services)	5	[36, 39, 40, 43, 49]
Chemical control (larvicide/adulticide)	5	[41, 51, 52, 53, 54]
Accommodation	3	[36, 46, 47]
Human resources (surveillance and integrated management)	3	[46, 53, 54]
Vector control	2	[36, 46]
Emergency actions (blocking activities)	2	[53, 54]
Health education/training/dissemination	2	[52, 53]
Nursing care	1	[36]
Entomological surveillance	1	[52]
Personal protective equipment	1	[54]

In India, Seyler et al. [44] estimated the cost of CHIKF in Mallela village, Andhra Pradesh state, from December 2005 to April 2006, reporting direct medical costs of US$ 7760 (US$ 32 per patient, 95% CI 25.8–38.1), which accounted for 86% of the total costs. Gopalan and Das [39] studied the familial impact of a CHIKF outbreak in Orissa, a state in Eastern India in 2007. They estimated that 86.3% of the CHIKF‐related costs were attributable to direct medical expenses, with a median per capita health expenditure of US$ 84, most of which was allocated to diagnostic procedures (US$ 77). Nearly 100% of participants incurred health expenditures exceeding 10% of household income. On average, catastrophic health expenditures accounted for 37% of household income.

In the same year, in Kerala state, India, Vijayakumar et al. [49] estimated an average of US$ 45.2 in out‐of‐pocket expenses incurred by patients. Of these, US$ 15.8 were direct costs, US$ 11.7 for medical (doctor's fees, medicine, and investigation charges), and US$ 4.1 for non‐medical (transportation, food, and others) expenses. Regarding catastrophic costs, approximately 30%–50% of families reported expenditures between 10% and 20% of household income, while more than 15% of families experienced expenditures exceeding twice their household income. The study concluded that direct healthcare expenses for families were high and exceeded the catastrophic health expenditures threshold, regardless of household income levels.

In Bolívar, a department located in the Caribbean region of Colombia, Alvis‐Zakzuk et al. [33] studied 126 clinically confirmed cases (67 children and 59 adults) during the first CHIKF outbreak in the country in 2014. The median direct medical cost for paediatric patients (US$ 257.9) was nearly four times higher than the estimate for adults (US$ 66.6). Hospitalisation costs comprised the largest share of direct medical costs for both paediatric (40.0%) and adult patients (38.1%), followed by laboratory and imaging costs (36.4% vs. 34.7%, respectively). The authors interviewed 15 adult patients to assess indirect costs and direct healthcare expenditures. In eight (53.3%) of the 15 households, income relied on one family member, and for nine of the 15 (60.0%), household income was below the minimum wage. An individual with CHIKF had an average direct expense of US$ 0.8 for transportation and US$ 4.2 for medications.

Also studying the 2014 CHIKF outbreak in Colombia, based on 106,592 confirmed cases, Cardona‐Ospina et al. [35] reported a total cost of at least US$ 73.6 million. Direct medical costs during the acute and post‐acute phase reached US$ 76.61, and during the chronic phase, estimates varied from US$ 1362.13 (the most conservative scenario) to US$ 3319.96 (the worst scenario).

In Dhaka, Bangladesh's capital, Hossain et al. [40] estimated direct expenditures during the outbreak's peak in 2017. Due to the absence of a national health insurance system in the country, the authors considered all treatment costs as out‐of‐pocket expenses for the patients, totalling US$ 99.3 for confirmed cases and US$ 29.6 for suspected cases. Furthermore, they found that the economic impact was more important for low‐income families (< US$ 303 per month).

In 2017, a study conducted in Fortaleza, Northeast Brazil, de Margarette et al. [36] estimated the direct medical costs of CHIKF, reporting a total healthcare expenditure of US$ 383,514.40 in a private hospital, of which US$ 174,322.91 (45.5%) was expended on emergency care and US$ 194,700.59 (50.8%) on hospitalisations. Additionally, the authors reported on 123 hospital professionals who were infected with CHIKV, generating an indirect cost of US$ 14,490.90 due to absenteeism from work. Also in Brazil, Gonçalves [38] estimated the cost of CHIKF for the Unified Health System (SUS, in Portuguese) in the municipality of Rio de Janeiro, where 37,904 CHIKF cases were registered in 2019. The total direct cost reached US$ 6,310,856.14, comprising costs in the acute (US$ 2,865,594.36), post‐acute (US$ 1,653,788.37) and chronic (US$ 1,721,287.69) phases, as well as hospitalisations (US$ 22,008.07) and diagnosis (US$ 48,177.65) costs.

#### 3.3.2 Indirect Costs: Productivity Loss

Sixteen studies estimated the indirect costs, resulting from absenteeism and reduced work performance due to the morbidity and mortality associated with CHIKF. The majority of these studies (80%) were published between 2009 and 2019, with original data from countries such as India [39, 42, 43, 44, 49, 50], Brazil [36, 38, 46] and Colombia [33, 35]. The number of workdays lost due to CHIKF varied widely across studies, ranging from one or at least 1 day [35, 49] to as many as 35 days [39] (Table 2).

In the Americas, Bloch [34] estimated a total of US$ 101.4 billion in indirect costs during the 2013–2015 CHIKV epidemic, accounting for 54.9% of total costs. The estimated economic losses at country level also varied significantly, ranging from US$ 8.5 million in India in 2006 [42] to US$ 38.5 million in Brazil in 2016 [46]. Overall, studies conducted in India [39], the United States [37], and one investigation incorporating data from the Netherlands, Sweden, Italy, Spain, and the United States [47] estimated that indirect costs related to productivity loss accounted for over 80% of the total cost of illness in those contexts.

Investigating the 2006 epidemic in India, Krishnamoorthy et al. [42] estimated the productivity loss to be a minimum of US$ 8.5 million. Nandha and Krishnamoorthy [43], studying data from the same epidemic, estimated an average of 11 (±6) workdays lost per patient due to CHIKF, costing US$ 7362.23 and with a corresponding average income loss of US$ 11.81 per individual. Furthermore, the study found that caregivers accompanying patients to healthcare facilities lost an average of 3.6 workdays, with a mean income loss of US$ 4.92. In Orissa state, India, Gopalan and Das [39] estimated a per‐patient cost of illness of US$ 88 in 2007, of which US$ 75 corresponded to productivity losses (35 median work days lost). In the same year, Vijayakumar et al. [49] reported that indirect costs reached US$ 29.4 in Kerala, India.

In 2014, Alvis‐Zakzuk et al. [33], studying 126 clinically confirmed CHIKF cases in Bolívar, Colombia, reported a median productivity loss of US$ 81.3 per adult patient. Cardona‐Ospina et al. [35], also investigating the 2014 CHIKF outbreak in Colombia, reported an indirect cost of US$ 60.2 per patient related to sick leave during the acute phase of the disease. From January to April 2015, Vázquez‐Cruz et al. [48] estimated the costs of disability due to CHIKF in Guerrero, Mexico, among 12,062 cases with 14,941 paid sick days, reaching a total indirect cost of US$ 152,505.36.

Tozan et al. [47] studied the costs related to international adult travellers returning to their home countries from 2016 to 2020 with malaria, dengue, CHIKF or Zika virus. The cases were predominantly from Africa (53%), followed by Asia (31%) and Central and South America (16%). The authors reported a median income loss of US$ 2400 associated with CHIKF.

In 2017, Margarette et al. [36] reported the indirect cost of CHIKF among 2683 patients in Fortaleza, Ceará state, reaching a total of US$ 14,490.90 due to absenteeism from work. More recently, in the municipality of Rio de Janeiro, Gonçalves [38] estimated the indirect cost due to CHIKF in 2019, reporting a total of US$ 26,970,132.70, of which 90.4% was related to the post‐acute and chronic phases (US$ 24,391,426.25).

### 3.4 Program Cost

Six studies [41, 46, 51, 52, 53, 54] reported program costs related to surveillance, prevention, and vector control. The national cost of vector control programmes was estimated by two studies, varying from US$ 888,000–1,476,300 annually in Greece (2013–2017) [41] to US$ 466 million in Brazil in 2016 [46]. In Machala, Ecuador, the median household expenses related to the acquisition of mosquito control products in areas infested with *Aedes aegypti* were US$ 2.00 per week (US$ 0–9.21) [51]. The study showed that families in low‐income communities spent more than 10% of their discretionary household income—that is, the amount remaining after expenses for essential household needs (e.g., food and shelter)—on interventions related to mosquito‐borne diseases.

Canali et al. [52] evaluated the expenditure on a vector control programme in the Emilia‐Romagna region (Northern Italy) during 2008–2011, reporting an annual expenditure of approximately US$ 1.63 per inhabitant, which decreased from US$ 2.97 in 2008 to US$ 1.75 in 2011.

Salinas‐López et al. [53], evaluating the vector‐borne disease control programmes in the municipalities (counties) of Girón and Guadalajara de Buga Colombia, in 2016, estimated total costs of US$ 146,651 (US$ 0.88 per capita) in Girón and US$ 97,936 (US$ 0.99 per capita) in Guadalajara de Buga, comprised mainly of expenses with personnel and chemical products.

In Yucatan State, Mexico, a cluster randomised trial was conducted during 2018–2019, aiming to quantify the entomological impact of preventive targeted indoor residual spraying in comparison with the reactive space spraying carried out by the Ministry of Health. They observed a reduction of 43%–70% in *Ae. aegypti* abundance in treatment houses compared to control houses, with an operational cost of US$ 4.2 to US$ 10.5 per house, depending on the insecticide cost [54].

### 3.5 Burden of Disease Studies

Ten of the 34 studies (29.4%) included in this review estimated the burden of CHIKF [34, 35, 37, 38, 42, 44, 46, 55, 56, 57] and were published between 2009 and 2022. Six of these employed a mixed‐methods approach, simultaneously analysing the cost and the burden of disease. Most studies were carried out in the region of the Americas (8/10), mainly in Brazil [38, 46, 55, 57] and Colombia [35, 56]. Five studies [34, 35, 42, 57, 76] presented estimates of DALYs per 100,000 population attributable to CHIKF, ranging from 4.53 in India (2006) to 2432 in the Region of the Americas (2013–2015) (Table 2).

The earliest published studies were conducted in India during the 2005–2006 outbreak, during which over 1.39 million suspected cases were recorded [31]. Krishnamoorthy et al. [42] used data from several Indian states in 2006 and estimated a burden of 25,588 DALYs lost, corresponding to 45.26 DALYs per million population. When comparing the burden of vector‐borne diseases in India, the authors found that the CHIKF burden was the lowest among the diseases analysed, including malaria, leishmaniasis, lymphatic filariasis, dengue and Japanese encephalitis. In contrast, a study conducted in Brazil by Teich et al. [46] showed that CHIKF accounted for the highest number of DALYs lost among other arboviruses (0.036 DALYs per episode of disease), such as yellow fever, dengue and Zika. In Colombia, using national data from 2013 to 2016, Mora‐Salamanca et al. [56] estimated that 71.3% of all DALYs attributable to arboviruses were due to CHIKF and post‐CHIKF chronic arthritis (350,531.62/491,629.2) DALYs. However, it is important to note that the result found by Krishnamoorthy et al. [42] may be significantly underestimated due to the high proportion of cases (78%) that used private healthcare networks, which were not reported in the public health information system.

In the region of the Americas, Bloch [34] analysed the burden of CHIKF from a macro‐regional perspective, showing a burden of 23.8 million DALYs (2432 DALYs per 100,000 population) during 2013–2015. Comparing the impact of CHIKF and dengue in the Americas between 2013 and 2015, the study showed that in just over 2 years of CHIKV‐induced epidemics, the annual total of DALYs lost due to CHIKF was 150 times greater than the estimated loss for dengue (72,277 DALYs annually). A similar finding was observed in the study by Seyler et al. [44], using data from the 2006 outbreak in India. Modelling the data for the worst‐case scenario, the authors estimated a national burden of 337,000 DALYs for CHIKF in India, exceeding the burden for dengue and Japanese encephalitis in 2002.

In Brazil, Vidal et al. [57] estimated the national burden of CHIKF in 2016–2017, reporting average values of 77,422.61 DALYs (0.3757 DALYs per 1000 inhabitants) for 2016, and 59,307.59 DALYs (0.2856 DALYs per 1000 inhabitants) for 2017. In both years studied, over 89% of the DALYs were attributed to the acute phase of the disease. Two other studies estimated the disease burden in Brazil at the local level, both in the municipality of Rio de Janeiro. Gonçalves [38] estimated a burden of CHIKF of 110.40 DALYs per 100,000 people in 2019, with an average of 0.3539 DALYs per case for the chronic phase of the disease. Between 2014 and 2019, Man et al. [55] estimated an annual burden of 2636 DALYs for CHIKF laboratory‐confirmed cases.

### 3.6 Cost‐Outcome Studies

Four of the 34 publications in this review were cost‐outcome studies of various preventive or control interventions for CHIKV and its vector. Three studies conducted cost‐effectiveness analyses [58, 59, 60], and one performed a cost‐utility analysis [61]. The studies were conducted in Colombia [58, 59] and Italy [60, 61] (Table 2).

Epidemiological mathematical models of the SIR and SEI‐SIR types were used. The SIR models the disease dynamics, including the human host population of *S*usceptible, *I*nfectious or Infected, and *R*ecovered individuals. The SEI‐SIR model incorporates new elements, such as the vectorial population (mosquitoes), into the SIR model. The latter model is particularly useful for vector‐borne diseases, where the interaction between these populations (human and vector) is critical for improving the understanding of disease transmission and the impact of environmental factors and disease control strategies such as those used for CHIKF. In addition to these models, other economic models were employed to estimate the epidemiological and economic impact of insecticide use in various contexts and assess the overall epidemiological and economic effects in different settings.

Guzzetta et al. [60] evaluated the epidemiological and economic impact of larvicide use in 10 municipalities in Northern Italy, focusing on preventing outbreaks in areas considered at moderate risk through a dynamic transmission model. The study concluded that routine larvicide‐based prevention in municipalities with fewer than 35,000 inhabitants effectively reduces mosquito populations, impacting the risk of CHIKV transmission and the magnitude of outbreaks triggered by imported cases in previously unaffected areas. A single, well‐timed larvicide application was projected to reduce local CHIKF transmission by 20% to 33%, with reductions reaching 43% to 65% if the treatment was repeated four times throughout the season. This strategy was associated with an estimated 0.45 DALYs averted and an average cost of US$ 515.9 per case prevented.

Trentini et al. [61] assessed the effectiveness of interventions using larvicides and adulticides for controlling *Aedes aegypti* at the onset of transmission (index case) in Italy. A stochastic mathematical model was employed to simulate the CHIKV transmission mechanism and the epidemiological conditions observed during the 2007 outbreak in the country. Model estimates indicated that had no intervention been implemented, the 2007 outbreak would have resulted in a total cost of US$ 20.1 million and an associated burden of 1600 DALYs. The deployment of insecticide during the outbreak was estimated to have generated cost savings of US$ 18.2 million in treatment‐related expenses.

In Colombia, studies conducted by Claypool et al. in 2019 and 2021 [58, 59] focused on evaluating the cost‐effectiveness and incremental benefits of indoor residual insecticide spraying in reducing clinical CHIKF cases and DALYs in endemic areas for both CHIKF and dengue. Modelling results suggested that insecticide use is a cost‐effective strategy and the preferred intervention for CHIKF and dengue prevention, with an incremental cost‐effectiveness ratio of US$ 3279 per DALY averted for CHIKF. The intervention was estimated to prevent 95 CHIKF cases per 100,000 population, compared to 67 cases per 100,000 population using insecticide‐treated bed nets. In a model comparing insecticide use with a hypothetical CHIKV vaccine, insecticide use was projected to avert 5390 DALYs, while the vaccine would avert 9954 DALYs. In a combined intervention model, insecticide use was associated with 12,375 DALYs averted, while the CHIKF vaccine would result in 11,808 DALYs averted.

### 3.7 Quality‐of‐Life Studies

Fifteen of the 43 studies included in the review (44.1%) evaluated the impact of CHIKF on patient functionality and QoL domains. These investigations were conducted between 2006 and 2020, mostly (5/15, 33.3%) in Brazil [62, 65, 66, 72, 75], followed by Curaçao [69, 70, 71] and India [67, 73]. Most studies focused on predominantly female populations, with participants aged 38–52. Although all included cases were confirmed through specific laboratory testing for CHIKF, considerable methodological variability was observed regarding the case definition criteria. Further details on the QoL studies are presented in Table 4.

**TABLE 4:** Characteristics of the included quality‐of‐life (QoL) studies.

Author (year of publication)	Location	Years studied and Scenario	Sample/population	Case definition	Assessment	Applied tool	Key findings
Barreto 2019 [62]	Brazil City of Fortaleza, Ceará State	November 2018 to August 2019 Public	42 participants 85.7% were female, with a mean age of 48 years	Cases reported and confirmed through laboratory testing	Post‐acute and chronic phases—Between 3‐ and 24‐months post‐infection	MEEM, WHODAS 2.0, WHO QoL‐bref	Mean pain intensity was 5.73 (±2.99). Mean functionality score was 35.55—domestic activity, participation and mobility domains were the most affected. The average QoL was 12.73 (±2.05), with environment and physical domains as the worst scores.
Couturier et al. 2012 [68]	France (mainland)	2005–2007 Public	391 participants (imported cases of CHIKV) 53.5% female, mean age 50.2 years 215 (55%) patients considered as not recovered	Patient with clinical symptoms of CHIKV infection confirmed by the presence of CHIKV‐specific IgM antibody or detection of CHIKV using RT‐PCR	Acute and chronic phases time (Median time from onset at follow‐up was 23.4 months)	SF‐36 AIMS2‐SF GHQ‐12 FCI	The AIMS2‐SF was affected mainly in symptoms, psychological and social dimensions. Scores of physical and mental components of the SF‐36 and GHQ‐12 were low. Older age was associated with lower SF‐36 scores. Other factors associated with lower SF‐36, lower GHQ12 scores and higher AIMS2‐SF dimensions were lack of recovery, presence of comorbidity and a longer duration of acute stage.
de Andrade et al. 2020 [63]	Réunion Island France	June to July 2006 Public	106 participants 74.5% were female, with a mean age of 47.3 years	Patients reporting pain with confirmed symptoms, and with previous serological confirmation of CHIKV infection (positive for IgG and IgM)	Chronic phase—1 year and 5 months (17 months) post‐infection	VAS BPI SF‐MPQ	51% presented with chronic pain. 18.9% reported pain with neuropathic characteristics (NP). Among patients with NP, 37% reported involvement of the lower limbs and 48% of the upper limbs. 53% experienced chronic pain. Total score of the SF‐MPQ and both the affective and sensory subscores were significantly higher in patients with NC. The mean pain interference in life activities calculated from the BPI was significantly higher in patients with chronic pain than in patients without it.
Doran et al. 2022 [69]	Curaçao, Netherlands (National)	2015–2017 Public	248 adult patients with chikungunya classified into the long‐term chikungunya disease severity categories: recovered, mildly affected, or highly affected Ratio of males to females was 0.37 51%—41 to 60 years	Adult patients with chikungunya and laboratory confirmed	Chronic phase—29 months after the disease onset	SF‐36	57% of the patients were still affected 2.5 years after disease onset. Patients highly affected reported an overall worsened QoL domains score, and worsened physical and mental scores, with 5.5 and 7.4 points change, respectively, in 2017 compared to 2015. Mildly affected patients reported a significant higher prevalence of non‐rheumatic symptoms skin diseases and sombreness in 2017 compared to 2015. Compared to mildly affected, being highly affected was associated with weakness in the lower extremities and worsened physical and mental QoL impairment.
Doran et al. 2022 [70]	Curaçao, Netherlands (National)	2019–2020 Public	304 patients were followed prospectively 169 (56%) patients were followed at all time points (74.6% female, mean age 56.1 years)	Laboratory confirmed patients	Chronic phase (3–16 months, 30 months and 60 months after disease onset)	SF‐36	107 (63%) were classified as recovered, and 62 (37%) as affected. 60 of the 62 affected (96.8%) reported experiencing arthralgia compared to 44 of the 107 (41.1%) recovered patients. Among the affected patients, 48.4% still experienced fatigue, 35.5% insomnia and 38.7% loss of vitality. Arthralgia in the upper and lower extremities, and headache were associated with being affected.
Elsinga et al. 2017 [71]	Curaçao, Netherlands (National)	2015 Public	304 adults, 74% were female, with age of 18–94 years. 36.2% of those clinically recovered 63.8% were defined as still being mildly (*n* = 105) or highly affected (*n* = 89) by chronic CHIKF	Laboratory confirmed patients	Chronic phase—(3–6 months) after diagnosis	SF‐36	At the time of interview, 63.8% (*n* = 194) were defined as still being mildly affected (*n* = 105, 34.5%) or highly affected (*n* = 89; 29.3%) by chronic chikungunya disease. Highly affected disease status was associated with clinical complaints (arthralgia, weakness, loss of vitality, and being diabetic) and major decreases in QoL scores.
Hayd et al. 2020 [72]	Brazil Roraima State	2017 Public	80 participants with a history of CHIKV infection were enrolled including 40 participants and 40 without persistent arthritis	Laboratory‐confirmed cases	Chronic phase (> 3 months post‐infection)	DAS28 EQ‐5D‐5L MSQ	Rheumatoid arthritis patients reported comparable quality of life measures in all five dimensions of the EQ‐5D except for a greater percentage reporting at least moderate effect on daily activities. CHIKV arthritis patients had a mean stiffness on the MSQ of 31.8% that was similar to findings in rheumatoid arthritis. Over 2 years post‐infection, patients report moderate arthritis disease severity comparable with rheumatoid arthritis with the most significant impact on decreased quality of life from pain.
Hossain et al. 2018 [40]	Bangladesh Dhaka City	2017 Public	1326 participants 57.2% were male, with a mean age of 33.7 years	Confirmed and probable cases diagnosed by physicians (82%)	Acute phase—During the first 2 weeks of infection	WHO QoL‐bref	Approximately 83% reported an overall poor to very poor quality of life. Older adults reported lower average QoL scores compared to those under 60 years of age. 85% of patients (both confirmed and probable cases) reported experiencing severe pain.
Jain et al. 2017 [73]	India Delhi and Mumbai	2010–2013 Public	572 participants 130 with arthralgia	Laboratory‐confirmed cases	Chronic phase—(> 3 months) post‐infection	VAS	56.14% reported restricted joint movement. intensity of their pain (VAS 1–10): the mean VAS was 6.05 with 34.6% patients had a VAS of 0–5 and 65.4% had a VAS of 6–10. Patients with higher viral load suffered severe chronic pain as substantiated by higher VAS (VAS > 5) irrespective of joint movement restrictions at the acute phase.
Marimoutou et al. 2015 [64]	Réunion Island France	2008 Public	252 participants 95% were male, with a mean age of 44 years	CHIK+ patients, including those with self‐reported CHIKV infection and those with positive serology (IgM and IgG)	Acute and chronic phases—6 years after initial infection	SF‐36 QoL scale	CHIK+ patients exhibited greater rheumatic morbidity. 48% reported moderate to severe pain. 48% experienced fatigue, 20% reported headaches, and 7% reported depressive mood. All quality‐of‐life domains were significantly compromised. 47% reported perceived difficulties in performing their work‐related activities. 46% were likely to report social disability.
Panato et al. 2019 [65]	Brazil Imperatriz City, Maranhão State	2017 Public and private	130 participants 92.3% were female, with a mean age of 52 years	Patients in the chronic phase of CHIKV infection, with laboratory‐confirmed diagnosis	Post‐acute phase—3 months after initial infection	RMDQ‐g	38.0% presented with functional disability. Patients with comorbidities and those who were on medication prior to infection exhibited greater functional impairment.
Paraense 2019 [66]	Brazil Belém City, Pará State	2016–2018 Public	65 participants 86.15% were female, with a mean age of 38.5 years	Patients who were conscious and oriented, with no verbal or cognitive impairments, and had a confirmed laboratory diagnosis	Acute and post‐acute phases—Approximately 3 months following initial infection	SF‐36	49% and 20% of participants reported experiencing moderate and severe pain, respectively. Physical and emotional aspects showed the highest levels of impairment (38.1 and 39, respectively). Pain (score: 43), vitality [48], and general health status (44.8) scores reflected notable limitations in these domains.
Ramachandran et al. 2012 [67]	India Chennai City, South India	2006 Public and private	403 participants (60%) were female; among them, 36% of those who were clinically non‐recovered were aged ≤ 35 years, while 62% of those clinically recovered were also aged ≤ 35 years	Patients diagnosed with CHIKV infection—Clinical Cases (C‐CHIKV)	Acute and chronic phases—Up to 5 months following initial infection	SF‐36 HRQoL scale	A more than 20‐fold reduction was observed in QoL scores in domains related to physical quality of life, as compared to healthy ‘normals’. A 3‐ to 5‐fold reduction was noted across other quality‐of‐life domains among C‐CHIKV patients who had not recovered, compared to healthy controls.
Simon et al. 2022 [74]	French Caribbean Island of Guadeloupe	2013–2015 Public	61 patients 51 females and 10 males, with a mean age of 62 years	Adults with persisting chronic symptoms after acute CHIKV infection, confirmed by IgM (ELISA) and/or indirect IgG ELISA	Chronic phase (36 months after chikungunya infection)	SF12	SF12 physical and mental component scores showed a poor health‐related quality of life. 86% reported that they stopped all physical activity. 68.4% reported anxiodepressive syndromes. 57.9% reported disturbed sleep at nights. 58.9% reported that they had to stop work for a period of at least 1 week during the chronic phase of the disease. Measures of joint pain, stiffness, and inflammation contributed to impaired quality of life scores. Fatigue and interrupted sleep appeared to be important predictors for physical aspects of quality of life.
Watson et al. 2021 [75]	Brazil Boa Vista, capital city of Roraima State	2019 Public	40 patients with chronic chikungunya arthritis 40 CHIKV negative patients with RA	Laboratory‐confirmed cases	Chronic phase—(two to 3 years after the outbreak)	HAQ EQ‐5D‐5L	The RA and CHIKV arthritis patients had similar symptom scores for pain intensity and stiffness severity, and similar levels of disability as measured by the HAQ. Stiffness was significantly associated with disability in chikungunya patients (anxiety and depression). Tender joint counts were significantly correlated with one quality of life domain (usual activity). Pain intensity score correlated with impaired mobility, self‐care and pain/discomfort domains.

Abbreviations: AIMS2‐SF—Short form of the Arthritis Impact Measurement Scales 2; BPI—Brief Pain Inventory; DAS28—Disease Activity Score in 28 joints; EQ‐5D‐5L—standardised measure of health status developed by the EuroQol Group; FCI—Functional Comorbidity Index; GHQ‐12—General Health Questionnaire; HAQ—Stanford Health Assessment Questionnaire; HRQoL—Health Related Quality of Life; MMSE—Mini‐Mental State Examination; MSQ—Musculoskeletal stiffness questionnaire; RMDQ‐g—Roland‐Morris Disability Questionnaire for general pain; SF‐12—12‐item short‐form health survey; SF‐36—36‐item short‐form health survey; SF‐MPQ—Short‐form McGill Pain Questionnaire; VAS—visual‐analogical scale; WHODAS 2.0—World Health Disability Assessment Schedule 2.0; WHOQoL‐bref—World Health Organization Quality of Life.

Regarding the timing of functionality and QoL assessments, the studies evaluated the following phases of the disease: acute [40]; acute and post‐acute [66]; acute and chronic [64, 68]; chronic [63, 69, 70, 71, 72, 73, 74, 75]; post‐acute and chronic [67] and post‐acute [65]. Seven of the 15 studies employed the Short Form Health Survey (SF‐36) [64, 66, 67, 68, 69, 70, 71]. Additionally, other studies adopted a variety of validated tools, including the Quality‐of‐Life Scale (QOLS) [64, 67]; Roland Morris Disability Questionnaire (RMDQ‐g) [65]; World Health Organization Disability Assessment Schedule 2.0 (WHODAS 2.0) [62] and the abbreviated version of the WHO Quality of Life questionnaire (WHOQoL‐bref) [40, 62].

The studies conducted in Brazil were primarily concentrated in the Northern region, specifically in the states of Roraima [72, 75], Pará [66] and Maranhão [65], from 2017 to 2019. Considerable heterogeneity was noted in the instruments used to assess QoL, thereby complicating direct comparisons across studies. The findings demonstrated marked impairments in the physical and mental domains. Furthermore, arthritis and pain associated with CHIKF were identified as contributors to greater functional impairment.

In Curaçao, an autonomous island state within the Kingdom of the Netherlands, three studies evaluated QoL in the same longitudinal prospective cohort at different time points: baseline (3–16 months following symptom onset) [71], first follow‐up (29–30 months) [69], and second follow‐up (60 months) [70]. The proportion of individuals affected by chronic CHIKF declined over time, from 63.8% at baseline to 57% at the first follow‐up, yet remained substantial (37%) even after 60 months of disease onset. At baseline, a highly affected disease status was associated with clinical manifestations such as arthralgia, weakness, loss of vitality and diabetes, alongside major reductions in QoL scores. By the 60‐month follow‐up, both the physical and mental component summary scores of affected individuals remained significantly lower and negatively associated when compared to CHIKV‐negative individuals.

In Réunion Island, Marimoutou et al. [64] reported that CHIKV‐infected patients exhibited more rheumatic morbidity compared to controls, with 48% of affected individuals reporting moderate to severe pain. De Andrade et al. [63], also investigating the CHIKF outbreak on Réunion Island, observed that 51% of participants experienced chronic pain, and nearly 20% reported pain with neuropathic characteristics. In this subgroup, the total score on the SF‐MPQ, and both the affective and sensory subscores, was significantly higher.

### 3.8 Methodological Quality Assessment of the Studies

Among the 25 cost‐of‐illness and burden‐of‐disease studies, 52% were classified as having medium‐high methodological quality [34, 35, 36, 37, 38, 39, 41, 42, 44, 47, 51, 53, 54], while 48% were rated as high quality [33, 40, 43, 45, 46, 48, 49, 50, 52, 55, 56, 57] (Table 7S). Of the four cost‐outcome studies, two were classified as having high methodological quality [59, 61], and two as medium‐high quality [58, 60] (Table 8S). Finally, all the QoL studies were considered to have high methodological quality [55, 56, 57, 62, 63, 64, 65, 66, 67, 68, 69, 70, 71, 72, 73, 74, 75] (Table 9S).

## 4 Discussion

As of March 2025 [77], CHIKV has been identified in over 110 countries and territories across Asia, Africa, Europe and the Region of the Americas. All regions with established populations of *Aedes aegypti* or *Aedes albopictus* mosquitoes have reported local CHIKV transmission via mosquito vectors [77, 78]. The majority of research on CHIKF has been conducted since the year 2000, with a substantial body of literature emerging in response to the 2005–2006 epidemic in Réunion Island and neighbouring islands in the Indian Ocean, as well as outbreaks in India and Southeast Asia beginning in 2006. In the Americas, CHIKV was first reported in December 2013 [79] and rapidly spread throughout the region, with Brazil becoming the epicentre of CHIKV epidemics in the Americas in 2016 [80].

Despite the increasing body of scientific literature on CHIKF and CHIKV, the still limited number of studies retrieved in this review (*n* = 43) does not appear to adequately reflect or document the disease's relevance in terms of its economic and social impact [8]. We acknowledge the existence of three previously published systematic reviews that addressed topics closely related to those examined in the present review. In 2017, van Aalst et al. [81] published a systematic literature review on long‐term sequelae of CHIKV infection, also investigating their impact on QoL. Their search was limited to the PubMed/MEDLINE database and included studies published in English between 2000 and 2016. The review identified five studies evaluating the impact of persistent manifestations on QoL, all of which were also captured in our search.

Costa et al. [82] investigated the global epidemiological and economic burden of CHIKF using four indexed databases (MEDLINE, Embase, LILACS and SciELO) and covering the period from 2007 to 2022. The authors identified only 13 economic evaluation studies on CHIKF, of which 10 were also included in our study. None of the three remaining publications met our inclusion criteria. Nonetheless, we managed to retrieve 23 publications on costs and burden of CHIKF that were not included by Costa et al. [82]. In addition to the search strategies and descriptors used—which may have limited the scope of retrieval—the review did not include grey literature beyond academic journals.

More recently, Tiozzo et al. [83] published a study comprising two systematic literature reviews. The first review focused on costs and resource utilisation, identifying 33 articles—16 related to costs and 17 addressing resource utilisation. Of the 16 cost‐related studies, two did not meet our inclusion criteria. Furthermore, the 17 studies exclusively addressing resource utilisation were not eligible for inclusion in our review. Notably, our systematic review identified 12 cost‐related studies that were not included in the review by Tiozzo et al. [83]. The second review was focused on QoL outcomes and retrieved 36 publications, of which 18 overlapped with those included in our review. The remaining 18 ineligible publications were categorised as follows: 9 scientific abstracts, 6 intervention studies employing QoL indicators, 2 studies that did not report QoL outcomes as defined in our review, and 1 brief report. It is important to note that the systematic reviews conducted by Tiozzo et al. [83] were not registered in PROSPERO, and the protocols are not publicly available. Additionally, the literature search was limited to the MEDLINE and Embase databases, and only publications in English were considered.

Overall, the limitations observed in those three systematic reviews have been comprehensively addressed and improved in the present study. In particular, the inclusion of a substantial number of additional studies—especially those published in languages other than English—has further strengthened the evidence base and contributed to more robust and comprehensive conclusions.

The rising number of CHIKF cases and their consequences have led to the designation of CHIKV as a priority pathogen and a threat to global public health, particularly in the context of climate change and globalisation [84]. It is estimated that more than half (51%) of individuals with laboratory‐confirmed symptomatic CHIKF experience chronic disability following infection [85]. Furthermore, although the rate of chronicity appears to decline over time, approximately one‐third of patients are estimated to remain functionally impaired 12 months post‐infection [86]. Thus, the clinical burden of the disease and its long‐term implications are likely to impose a substantial economic burden on affected households, health systems and local and national economies.

CHIKF has increasingly surpassed the economic impact of other, longer known and prevalent arboviral diseases such as dengue and yellow fever, as well as other infectious diseases, including HIV/AIDS and tuberculosis—particularly in low‐ and middle‐income countries across the Americas, Africa and Asia. In 2014, the chronic burden associated with CHIKV in Latin America, as measured in DALYs attributable to the virus, exceeded that of any other arbovirus in the region for that year [35]. Between 2010 and 2019, CHIKV was estimated to be responsible for an average annual loss of over 106,000 DALYs globally—substantially higher than that attributed to ZIKV (44,000 DALYs). In the Americas, CHIKF may have caused an average annual loss of more than 158,000 DALYs since its emergence, underscoring CHIKV as one of the most burdensome arboviruses in the region [8].

The majority of the publications included in this review originate from countries classified by the World Bank as lower middle‐income (India, with a per capita income of US$ 1145 or less) and upper‐middle‐income (Brazil, Colombia and Mexico, with per capita incomes ranging from US$ 1146 to US$ 4515). These countries are characterised by mixed health systems (public and private), although with markedly different configurations. India, for instance, maintains a predominantly private healthcare system supplemented by targeted programmes for vulnerable populations. In contrast, Brazil operates a Unified Health System (Sistema Único de Saúde, in Portuguese), which provides free access to all citizens and coexists with a parallel private sector. In such settings, estimating the actual costs associated with CHIKF remains challenging due to limitations in surveillance quality, as highlighted in the scoping review by Mascarenhas et al. [16].

Direct and indirect costs can exacerbate affected families' economic and financial situation, particularly in developing countries. Losses associated with morbidity and productivity may be even higher when considering the expenses of family members who generally accompany the patients, especially in cases involving children or the elderly. Furthermore, healthcare expenses directly impact the family economy and, consequently, the broader society. The greater the healthcare expenditure the patient covers, the lower the family's well‐being regarding healthcare access and living standards, and a significant cause of impoverishment in the affected communities [25, 87].

The partial economic evaluation approach was employed to estimate the costs of CHIKF in most of the studies included in this review. These ‘disease cost’ and ‘program cost’ analyses indicate the amount that would have been saved in the absence of the disease, which could have been allocated to other expenses or investments [76, 88]. A significant diversity of methodologies, analytic perspectives and indicators was observed, with studies estimating direct costs, especially medical costs, and indirect costs, including productivity losses. Thus, the adoption of standardised economic evaluation frameworks for assessing the impact of CHIKF across diverse geographical and healthcare settings is essential to generate robust and comparable evidence on its economic burden. Notably, all studies addressing the indirect costs of CHIKF employed the human capital method, which uses wages to estimate productivity costs by considering the number of days absent from work and daily wage values [20, 89], assuming full employment scenarios. These studies demonstrated that productivity losses accounted for a significantly large proportion of the total costs associated with CHIKF.

Estimates of the burden of disease, using the DALY indicator or one of its components (YLL or YLD), may be underestimated in the studies retrieved, as they are directly influenced by factors such as passive surveillance, applied in most contexts rather than active case finding; the absence of more robust data regarding the over 40% of estimated cases that progress to chronic disease [90]; and the scarcity of information on hospitalisation or severe cases, mortality and lethality, which may explain differences in the findings of the studies reviewed [82].

In this scenario, our results reinforce the need for longitudinal cohort studies that systematically assess chronic outcomes and long‐term disability associated with CHIKF, which are essential to accurately quantify the burden of the disease. Robust estimates of chronic morbidity and DALYs would provide a more comprehensive understanding of the enduring health impacts of CHIKF and are critical to informing national and global health priorities. Furthermore, the routine inclusion of CHIKF in global burden of disease (GBD) assessments is urgent to document its growing epidemiological and socioeconomic impact, particularly in regions with recurrent outbreaks. Without these efforts, the true burden of CHIKF will continue to be underestimated, limiting the evidence base for resource allocation, public health planning and prioritisation of research and intervention strategies.

Additionally, we highlight the lack of a defined disability weight for CHIKF and its chronic sequelae, resulting in heterogeneity of this component/value, requiring the derivation of the disability weight for CHIKF from other diseases. The disability weight is a measure that ranges from 0 to 1, where 0 represents a healthy life and 1 represents death. For the acute phase of the disease, the analysed studies adopted weights ranging from 0.051 (moderate dengue) to 0.133 (severe infectious disease). In the chronic phase, most studies (> 60%) adopted the disability weight estimated for rheumatoid arthritis (0.233).

Complementarily, the findings of this review corroborate evidence from studies that highlight the potential and the cost‐effectiveness and cost‐utility relationship of the timely use of larvicides and adulticides in reducing mosquito population density and, consequently, reducing the risk of contracting the disease [16]. However, while it is presumed that reducing mosquito populations would decrease transmission risk, few studies provide conclusive evidence of this relationship [91].

Some limitations must be considered for the appropriate interpretation of the findings of this review. First, considering the methodology used, it is possible that the search strategy did not identify some publications or were not retrieved due to language limitations. However, it is important to emphasise that the study was conducted using a rigorous and reproducible methodology, including the review of grey literature, particularly in Brazil, through searches in thesis and dissertation databases. Second, the data reported on CHIKF mainly come from outbreaks and epidemics, which hampers the establishment of standardised and rigorous scientific protocols, as researchers need to adapt to the dynamic nature of the event. Moreover, most of the data are analysed retrospectively, sometimes hindering the accurate estimation of specific indicators. Thus, significant heterogeneity is observed in the methods and the estimates presented, resulting in a limited number of studies employing comparable methodologies and metrics. Third, given the aforementioned heterogeneity, it was impossible to consolidate estimates from different studies by applying meta‐analysis techniques.

Finally, we acknowledge certain limitations inherent in the tools employed to assess the methodological quality of the studies, particularly regarding their ability to detect potential biases in cost estimation. These tools can offer limited appraisal of the validity, accuracy and representativeness of the cost data sources used, as well as insufficient assessment of the appropriateness of the costing methods applied, which can vary substantially across studies and introduce methodological heterogeneity. These limitations indicate that, while structured quality assessment tools offer valuable guidance, they may not comprehensively capture the potential for bias specifically related to cost estimation in economic evaluations.

In summary, this systematic review reported the key and most relevant publications in the literature addressing the economic and social impact of CHIKF, emphasising the importance of studies aimed at estimating the economic and social burden of the disease, including direct healthcare costs caused by CHIKF, as well as other costs for patients and families, such as expenses with repellents. These findings reinforce the understanding of CHIKF as a disease with chronic impacts throughout an individual's life, affecting various dimensions beyond health, leading to absenteeism, economic losses and years of life lost. Furthermore, the consequences of the disease impact healthcare systems and economies at both national and local levels, depending on the scope of outbreaks and epidemics.

The limitations in knowledge regarding the cost and burden of CHIKF are related to inadequate surveillance and underdiagnosis, especially in scenarios of co‐circulation and coinfection with CHIKV, DENV and ZIKV. In this context, the importance of early detection and appropriate management of CHIKF is highlighted, as these measures can prevent severe cases related to hospitalisation and mortality, as well as long‐term consequences such as arthritis and chronic joint pain, which result in loss of productivity and reduced QoL for the affected individuals. Additionally, there is a need for standardised methods to minimise the inherent limitations in economic evaluations, such as the design of the health intervention or programme being assessed, seeking to overcome epidemiological and demographic differences, distinct clinical practices and financing methods between regions and countries for economic evaluation and burden estimation of CHIKF. Thus, it is hoped that a robust body of evidence will be generated to support the proposal, implementation and evaluation of public policies that support individuals affected by the disease, both in healthcare and social assistance, especially in middle‐ or low‐income countries and vulnerable contexts.

## Conflicts of Interest

The authors declare no conflicts of interest.

## Supporting information


Data S1.
