# Supplementary material for: Burden of Chikungunya Fever and Its Economic and Social Impacts Worldwide: A Systematic Review
Source: Trop Med Int Health. 2025 Jul 22;30(9):865–92. doi: 10.1111/tmi.70012 (PMC12401649; doi:10.1111/tmi.70012)
Supplement: Supplementary file 1 — Data S1. [file TMI-30-865-s001.docx]

**SUPPLEMENTARY MATERIAL**

Table 1S - Outline of the search strategy according to the research question constructed from PICOT

| **Acronym/Description** | **Definition** |
| --- | --- |
| **P** (Population/Patient) | Individuals suspected or diagnosed with Chikungunya fever |
| **I/E** (Exposure) | **---** |
| **C** (Comparator) | **---** |
| **O** (Outcomes) | Disability-Adjusted Life Years (DALYs)  Quality-Adjusted Life Years (QALYs)  Years of Life Lost due to premature mortality (YLLs)  Years Lived with Disability (YLDs)  Direct medical and non-medical costs  Indirect costs  Catastrophic health expenditures  Economic losses |
| **T** (Study Types) | Health economic evaluation studies  Cost-of-illness studies  Program cost studies  Burden-of-disease studies  Qualitative studies assessing social and economic losses |

Table 2S - Keywords/Search terms used to develop the search strategy in each database

| Database | Keywords/Search terms |
| --- | --- |
| **Medline** | Chikungunya Fever, Disability-Adjusted Life Years, Quality-Adjusted Life Years, Costs and Cost Analysis, Health Care Costs, Direct Service Costs, Cost-Benefit Analysis, Cost of Illness, Health Expenditures |
| **Embase** | 'chikungunya'/exp, 'disability - adjusted life year'/exp, 'daly'/exp, 'dalys'/exp, 'quality adjusted life year'/exp, 'qaly'/exp , 'cost'/exp , 'cost allocation'/exp, 'cost sharing'/exp, 'deductibles and coinsurance'/exp, 'costs and cost analysis'/exp, 'health care cost'/exp, 'capital expenditures'/exp, 'employer health costs'/exp, 'health care costs'/exp, 'health care economics'/exp, 'health care economics and organisations'/exp, 'health care expenditure'/exp, 'health care sector'/exp, 'healthcare cost'/exp, 'healthcare economics'/exp, 'healthcare expenditure'/exp, 'healthcare sector'/exp, 'direct service costs'/exp, 'health expenditures'/exp, 'economic evaluation'/exp , 'cost benefit analysis'/exp, 'cost analysis'/exp , 'cost benefit'/exp, 'cost benefit ratio'/exp, 'cost-benefit analysis'/exp, 'cost minimization analysis'/exp , 'cost minimization'/exp , 'cost effectiveness analysis'/exp, 'cost effectiveness'/exp, 'cost effectiveness ratio'/exp , 'cost efficiency analysis'/exp, 'cost utility analysis'/exp, 'cost utility'/exp, 'cost of illness'/exp, 'cost of illness analysis'/exp , 'economic aspects of illness'/exp, 'catastrophic health expenditure'/exp |
| **Lilacs** | "Febre de Chikungunya", “Anos de Vida Ajustados pela Incapacidade”, "Anos de Vida Ajustados por Qualidade de Vida", "Custos e Análise de Custo", "Custos de Cuidados de Saúde", "Custos Diretos de Serviços", "Análise Custo-Benefício”, "Análise Custo-Benefício”, "Efeitos Psicossociais da Doença”, “Gastos em Saúde", "Gasto Catastrófico em Saúde" |

**Table 3S -** Syntax for searching articles in the MEDLINE database (accessed via PubMed)

| **Syntax 1- MEDLINE** |
| --- |
| (Chikungunya Fever[MeSH Terms]) OR ((Chikungunya Fever) OR (Chikungunya Fevers) OR (Fever, Chikungunya) OR (Chikungunya Virus Infection) OR (Chikungunya Virus Infections) OR (Infection, Chikungunya Virus) OR (Chickungunya Fever) OR (Chickungunya Fevers) OR (Fever, Chickungunya)))) AND ((((((((((Disability-Adjusted Life Years [MeSH Terms]) OR ((Disability-Adjusted Life Years) OR (Disability Adjusted Life Years) OR (Disability-Adjusted Life Year) OR (Life Year, Disability-Adjusted) OR (Life Years, Disability-Adjusted) OR (DALYs) OR (Years Lived With Disability) OR (YLDs) OR (Years of life lost) OR (YLL))) OR ((Quality-Adjusted Life Years[MeSH Terms]) OR ((Quality-Adjusted Life Years) OR (Life Year, Quality-Adjusted) OR (Life Years, Quality-Adjusted) OR (QUALYs) OR (QUALY) OR (Quality Adjusted Life Years) OR (Quality Adjusted Life Year) OR (Quality-Adjusted Life Year) OR (QALYs) OR (Healthy Years Equivalents) OR (Healthy Years Equivalent) OR (Equivalents, Healthy Years) OR (Adjusted Life Years) OR (Adjusted Life Year) OR (Life Year, Adjusted) OR (Life Years, Adjusted) OR (Years, Adjusted Life)))) OR ((Costs and Cost Analysis[MeSH Terms]) OR ((Costs and Cost Analysis) OR (Costs, Cost Analysis) OR (Cost, Cost Analysis) OR (Costs and Cost Analyses) OR (Cost Analysis) OR (Analysis, Cost) OR (Analyses, Cost) OR (Cost Analyses) OR (Cost Comparison) OR (Comparison, Cost) OR (Comparisons, Cost) OR (Cost Comparisons) OR (Affordability) OR (Affordabilities) OR (Cost-Minimization Analysis) OR (Analyses, Cost-Minimization) OR (Analysis, Cost-Minimization) OR (Cost Minimization Analysis) OR (Cost-Minimization Analyses) OR (Pricing) OR (Cost) OR (Costs) OR (Cost Measures) OR (Cost Measure) OR (Measure, Cost) OR (Measures, Cost)))) OR ((Health Care Costs[MeSH Terms]) OR ((Health Care Costs) OR (Cost, Health Care) OR (Costs, Health Care) OR (Health Care Cost) OR (Health Costs) OR (Cost, Health) OR (Costs, Health) OR (Health Cost) OR (Healthcare Costs) OR (Cost, Healthcare) OR (Costs, Healthcare) OR (Healthcare Cost) OR (Medical Care Costs) OR (Costs, Medical Care) OR (Cost, Medical Care) OR (Medical Care Cost) OR (Treatment Costs) OR (Cost, Treatment) OR (Costs, Treatment) OR (Treatment Cost)))) OR ((Direct Service Costs[MeSH Terms]) OR ((Direct Service Costs) OR (Costs, Direct Service) OR (Service Costs, Direct) OR (Cost, Direct Service) OR (Direct Service Cost) OR (Service Cost, Direct)))) OR ((Cost-Benefit Analysis[MeSH Terms]) OR ((Cost-Benefit Analysis) OR (Analyses, Cost-Benefit) OR (Analysis, Cost-Benefit) OR (Cost-Benefit Analyses) OR (Cost Benefit Analysis) OR (Analyses, Cost Benefit) OR (Analysis, Cost Benefit) OR (Cost Benefit Analyses) OR (Cost Effectiveness) OR (Effectiveness, Cost) OR (Cost-Benefit Data) OR (Cost Benefit Data) OR (Data, Cost-Benefit) OR (Cost-Utility Analysis) OR (Analyses, Cost-Utility) OR (Analysis, Cost-Utility) OR (Cost Utility Analysis) OR (Cost-Utility Analyses) OR (Economic Evaluation) OR (Economic Evaluations) OR (Evaluation, Economic) OR (Evaluations, Economic) OR (Marginal Analysis) OR (Analyses, Marginal) OR (Analysis, Marginal) OR (Marginal Analyses) OR (Cost Benefit) OR (Costs and Benefits) OR (Benefits and Costs) OR (Cost and Benefit) OR (Benefit and Cost) OR (Cost-Effectiveness Analysis) OR (Analysis, Cost-Effectiveness) OR (Cost Effectiveness Analysis)))) OR ((Cost of Illness[MeSH Terms]) OR ((Cost of Illness) OR (Illness Cost) OR (Illness Costs) OR (Cost of Sickness) OR (Sickness Costs) OR (Sickness Cost) OR (Burden of Illness) OR (Illness Burden) OR (Illness Burdens) OR (Disease Burden) OR (Burden, Disease) OR (Disease Burdens) OR (Costs of Disease) OR (Disease Cost) OR (Cost, Disease) OR (Disease Costs) OR (Economic Burden of Disease) OR (Burden Of Disease) OR (Burden Of Diseases) OR (Cost of Disease)))) OR ((Health Expenditures[MeSH Terms]) OR ((Health Expenditures) OR (Expenditure, Health) OR (Health Expenditure) OR (Expenditures, Health) OR (Expenditures, Direct) OR (Direct Expenditure) OR (Direct Expenditures) OR (Expenditure, Direct) OR (Expenditures, Out-of-Pocket) OR (Expenditure, Out-of-Pocket) OR (Expenditures, Out of Pocket) OR (Out-of-Pocket Expenditure) OR (Out-of-Pocket Expenditures) OR (Out-of Pocket Expenditures) OR (Expenditure, Out-of Pocket) OR (Expenditures, Out-of Pocket) OR (Out of Pocket Expenditures) OR (Out-of Pocket Expenditure) OR (Out-of-Pocket Expenses) OR (Out of Pocket Expenses) OR (Out-of-Pocket Costs) OR (Out of Pocket Costs) OR (Out-of-Pocket Expense) OR (Expense, Out-of-Pocket) OR (Expenses, Out-of-Pocket) OR (Out of Pocket Expense) OR (Out-of-Pocket Payments) OR (Out of Pocket Payments) OR (Out Of Pocket Expenditure) OR (Out-of-Pocket Cost) OR (Cost, Out-of-Pocket) OR (Costs, Out-of-Pocket) OR (Out of Pocket Cost) OR (Out-of-Pocket Payment) OR (Out of Pocket Payment) OR (Payment, Out-of-Pocket) OR (Payments, Out-of-Pocket) OR (Out-of-Pocket Spending) OR (Out of Pocket Spending) OR (Spending, Out-of-Pocket) OR (Expenditures) OR (Expenditure) OR (Expenditures, Indirect) OR (Expenditure, Indirect) OR (Indirect Expenditure) OR (Indirect Expenditures)))) OR ((Economics Losses) OR (Catastrophic Costs) OR (Catastrophic Health Expenditure) OR (Social Sequels))) |

**Table 4S -** Syntax for searching articles in the EMBASE database (accessed via the CAPES portal)

| **Syntax 2- EMBASE (CAPES)** |
| --- |
| ('chikungunya'/exp OR 'chikungunya' OR 'chickungunya'/exp OR 'chickungunya' OR 'chikungunya disease'/exp OR 'chikungunya disease' OR 'chikungunya fever'/exp OR 'chikungunya fever' OR 'chikungunya virus infection'/exp OR 'chikungunya virus infection') AND ('disability-adjusted life year'/exp OR 'disability-adjusted life year' OR 'daly'/exp OR 'daly' OR 'dalys'/exp OR 'dalys' OR 'disability-adjusted life years'/exp OR 'disability-adjusted life years' OR 'quality adjusted life year'/exp OR 'quality adjusted life year' OR 'qaly'/exp OR 'qaly' OR 'quality adjusted life years'/exp OR 'quality adjusted life years' OR 'quality-adjusted life years'/exp OR 'quality-adjusted life years' OR 'cost'/exp OR 'cost' OR 'cost allocation'/exp OR 'cost allocation' OR 'cost sharing'/exp OR 'cost sharing' OR 'deductibles and coinsurance'/exp OR 'deductibles and coinsurance' OR 'costs and cost analysis'/exp OR 'costs and cost analysis' OR 'health care cost'/exp OR 'health care cost' OR 'capital expenditures'/exp OR 'capital expenditures' OR 'employer health costs'/exp OR 'employer health costs' OR 'health care costs'/exp OR 'health care costs' OR 'health care economics'/exp OR 'health care economics' OR 'health care economics and organisations'/exp OR 'health care economics and organisations' OR 'health care economics and organizations'/exp OR 'health care economics and organizations' OR 'health care expenditure'/exp OR 'health care expenditure' OR 'health care sector'/exp OR 'health care sector' OR 'healthcare cost'/exp OR 'healthcare cost' OR 'healthcare economics'/exp OR 'healthcare economics' OR 'healthcare expenditure'/exp OR 'healthcare expenditure' OR 'healthcare sector'/exp OR 'healthcare sector' OR 'direct service costs'/exp OR 'direct service costs' OR 'health expenditures'/exp OR 'health expenditures' OR 'economic evaluation'/exp OR 'economic evaluation' OR 'cost benefit analysis'/exp OR 'cost benefit analysis' OR 'cost analysis'/exp OR 'cost analysis' OR 'cost benefit'/exp OR 'cost benefit' OR 'cost benefit ratio'/exp OR 'cost benefit ratio' OR 'cost-benefit analysis'/exp OR 'cost-benefit analysis' OR 'cost minimization analysis'/exp OR 'cost minimization analysis' OR 'cost minimization'/exp OR 'cost minimization' OR 'cost effectiveness analysis'/exp OR 'cost effectiveness analysis' OR 'cost effectiveness'/exp OR 'cost effectiveness' OR 'cost effectiveness ratio'/exp OR 'cost effectiveness ratio' OR 'cost efficiency analysis'/exp OR 'cost efficiency analysis' OR 'cost utility analysis'/exp OR 'cost utility analysis' OR 'cost utility'/exp OR 'cost utility' OR 'cost of illness'/exp OR 'cost of illness' OR 'cost of illness analysis'/exp OR 'cost of illness analysis' OR 'economic aspects of illness'/exp OR 'economic aspects of illness' OR 'economics losses' OR 'catastrophic costs' OR 'catastrophic health expenditure'/exp OR 'catastrophic health expenditure' OR 'social sequels') |

**Table 5S -** Syntax for searching articles in the LILACS database (accessed via BVS)

| **Syntax 3 - LILACS (BVS)** |
| --- |
| ((mh:"Febre de Chikungunya") OR ((Febre de Chikungunya) OR (Chikungunya Fever) OR (Fiebre Chikungunya) OR (Fièvre chikungunya) OR (Chicungunha) OR (Chikungunya) OR (Febre Chickungunya) OR (Febre Chikungunya) OR (Febre do Chikungunya) OR (Infecção pelo Vírus Chikungunya) OR (Infecção por Vírus Chikungunya))) AND (((mh:"Anos de Vida Ajustados pela Incapacidade") OR ((Anos de Vida Ajustados pela Incapacidade) OR (Disability-Adjusted Life Years) OR (Años de vida Ajustados por la Incapacidad) OR (Années de Vie Ajustées Au Handicap) OR (AVPI) OR (Anos de Vida Perdidos por Incapacidade))) OR ((mh:"Anos de Vida Ajustados por Qualidade de Vida") OR ((Anos de Vida Ajustados por Qualidade de Vida) OR (Quality-Adjusted Life Years) OR (Años de Vida Ajustados por Calidad de Vida) OR (Années de vie ajustées sur la qualité) OR (AVAQ) OR (Ano de Vida Ajustado) OR (Ano de Vida Ajustado pela Qualidade) OR (Ano de Vida Ajustado pela Qualidade de Vida) OR (Ano de Vida Ajustado por Qualidade de Vida) OR (Anos de Vida Ajustados por Qualidade de Vida) OR (AVAQ) OR (Equivalente em Anos Saudáveis) OR (QALY) OR (Qualidade dos Anos de Vida Padronizados))) OR ((mh:"Custos e Análise de Custo") OR ((Custos e Análise de Custo) OR (Costs and Cost Analysis) OR (Costos y Análisis de Costo) OR (Coûts et analyse des coûts) OR (Acessibilidade Financeira) OR (Análise de Custo em Saúde) OR (Análise de Custos) OR (Análise de Minimização de Custo) OR (Análise de Minimização de Custos) OR (Capacidade Aquisitiva) OR (Capacidade de Pagar pelos Serviços de Saúde) OR (Capacidades de Pagar pelos Serviços de Saúde) OR (Comparação de Custos) OR (Custo) OR (Custos) OR (Custos e Análises de Custo) OR (Medidas de Custo) OR (Precificação))) OR ((mh:"Custos de Cuidados de Saúde") OR ((Custos de Cuidados de Saúde) OR (Health Care Costs) OR (Costos de la Atención en Salud) OR (Coûts des soins de santé) OR (Custos de Cuidados Médicos) OR (Custos de Tratamento))) OR ((mh:"Custos Diretos de Serviços") OR ((Custos Diretos de Serviços) OR (Direct Service Costs) OR (Costos Directos de Servicios) OR (Coûts directs des services) OR (Custo Direto))) OR ((mh:"Análise Custo-Benefício") OR ((Análise Custo-Benefício) OR (Cost-Benefit Analysis) OR (Análisis Costo-Beneficio) OR (Analyse coût-bénéfice) OR (Análise de Custo-Benefício) OR (Análise de Custo-Efetividade) OR (Análise de Custo-Utilidade) OR (Custo-Efetividade) OR (Dados de Custo-Benefício))) OR ((mh:"Efeitos Psicossociais da Doença") OR ((Efeitos Psicossociais da Doença) OR (Cost of Illness) OR (Costo de Enfermedad) OR (Coûts indirects de la maladie) OR (Carga da Doença) OR (Carga das Doenças) OR (Carga de Doença) OR (Custo da Doença) OR (Custo da Doença para o Paciente) OR (Custos da Doença) OR (Fardo da Doença) OR (Fardos Relativos à Doença) OR (Peso da Doença) OR (Ônus da Doença))) OR ((mh:"Gastos em Saúde") OR ((Gastos em Saúde) OR (Health Expenditures) OR (Gastos en Salud) OR (Dépenses de santé) OR (Contas Nacionais de Saúde) OR (Custeio) OR (Despesa) OR (Despesa com Saúde) OR (Despesa do Bolso) OR (Despesas) OR (Despesas Diretas) OR (Despesas Indiretas) OR (Despesas com Saúde) OR (Despesas de Bolso) OR (Despesas em Saúde) OR (Gasto) OR (Gasto com Saúde) OR (Gasto de Bolso) OR (Gastos) OR (Gastos Diretos) OR (Gastos Indiretos) OR (Gastos com Saúde) OR (Gastos de Bolso) OR (Pagamento Direto))) OR ((mh:"Gasto Castrófico em Saúde") OR (mh:"Gasto Castrófico em Saúde" (Gasto Castrófico em Saúde) OR (Catastrophic Health Expenditure) OR (Gasto Catastrófico en Salud) OR (Dépenses de Santé Catastrophiques) OR (Gasto Catastrófico))) OR ((Perdas econômicas) OR (Sequelas Sociais))) |

**Table 6S** – Studies selected for systematic review, according to type of evaluation conducted

| **Author/Year (Ref)** | **Title** | **Available at:** |
| --- | --- | --- |
| **Cost-of-Illness and Burden-of-Disease studies** | | |
| Alvis-Zakzuk *et al.* 2018 (33) | Economic Costs of Chikungunya Virus in Colombia | <https://pubmed.ncbi.nlm.nih.gov/29627722/> |
| Bloch 2016 (34) | The Cost and Burden of Chikungunya in The Americas (2016) | <https://elischolar.library.yale.edu/ysphtdl/1022/> |
| Canali *et al.* 2017 (52) | The Cost of Arbovirus Disease Prevention in Europe: Area-Wide Integrated Control of Tiger Mosquito, *Aedes albopictus*, in Emilia-Romagna, Northern Italy | <https://www.ncbi.nlm.nih.gov/pmc/articles/PMC5409644/> |
| Cardona-Ospina *et al.* 2015 (35) | Estimating the burden of disease and the economic cost attributable to chikungunya, Colombia, 2014 | https://pubmed.ncbi.nlm.nih.gov/26626342/ |
| De Margarette *et al.* 2022 (36) | Chikungunya in Brazil: An epidemic of high cost for private healthcare, 2017 | <https://pubmed.ncbi.nlm.nih.gov/36054244/> |
| Feldstein *et al.* 2019 (37) | Estimating the cost of illness and burden of disease associated with the 2014–2015 chikungunya outbreak in the U.S. Virgin Islands | <https://pubmed.ncbi.nlm.nih.gov/31323020/> |
| Gonçalves 2021 (38) | Os custos da Chikungunya no município do Rio de Janeiro e sua relação com variáveis climáticas | <https://www.arca.fiocruz.br/handle/icict/55925> |
| Gopalan & Das 2009 (39) | Household economic impact of an emerging disease in terms of catastrophic out-of-pocket health care expenditure and loss of productivity: investigation of an outbreak of chikungunya in Orissa, India | <https://pubmed.ncbi.nlm.nih.gov/19326709/> |
| Heydari *et al.* 2017 (51) | Household Dengue Prevention Interventions, Expenditures, and Barriers to *Aedes aegypti* Control in Machala, Ecuador | <https://pubmed.ncbi.nlm.nih.gov/28212349/> |
| Hossain *et al.* 2018 (40) | Chikungunya outbreak (2017) in Bangladesh: Clinical profile, economic impact and quality of life during the acute phase of the disease | https://pubmed.ncbi.nlm.nih.gov/29874242/ |
| Kaur *et al.* 2022 (50) | Economic burden estimation associated with dengue and chikungunya in Gujarat, India | <https://pmc.ncbi.nlm.nih.gov/articles/PMC9731033/> |
| Kolimenakis *et al.* 2019 (41) | On lifestyle trends, health and mosquitoes: Formulating welfare levels for control of the Asian tiger mosquito in Greece | <https://pubmed.ncbi.nlm.nih.gov/31163025/> |
| Krishnamoorthy *et al.* 2009 (42) | Burden of chikungunya in India: Estimates of Disability Adjusted Life Years (DALY) lost in 2006 epidemic | https://pubmed.ncbi.nlm.nih.gov/19326705/ |
| Man *et al.* 2022 (55) | Re-emergence of arbovirus diseases in the State of Rio de Janeiro, Brazil: The role of simultaneous viral circulation between 2014 and 2019 | <https://pubmed.ncbi.nlm.nih.gov/36277093/> |
| Mora-Salamanca *et al.* 2020 (56) | Estimating the burden of arboviral diseases in Colombia between 2013 and 2016 | <https://pubmed.ncbi.nlm.nih.gov/32434085/> |
| Nandha & Krishnamoorthy 2009 (43) | Cost of Illness due to Chikungunya during 2006 Outbreak in a Rural Area in Tamil Nadu | <https://pubmed.ncbi.nlm.nih.gov/20469756/> |
| Salinas-López *et al.* 2018 (53) | Costos de un programa de control del vector *Aedes aegypti* en municipios de Colombia: el caso de Girón y Guadalajara de Buga, 2016 | <https://search.bvsalud.org/portal/resource/pt/biblio-974610> |
| Seyler *et al.* 2010 (44) | Estimating the burden of disease and the economic cost attributable to chikungunya, Andhra Pradesh, India, 2005–2006 | <https://pubmed.ncbi.nlm.nih.gov/19709705/> |
| Soumahoro *et al.* 2011 (45) | The Chikungunya Epidemic on La Reunion Island in 2005–2006: A Cost-of-Illness Study | https://pubmed.ncbi.nlm.nih.gov/21695162/ |
| Teich *et al.* 2017 (46) | *Aedes aegypti* e sociedade: O impacto econômico das arboviroses no Brasil | <https://pesquisa.bvsalud.org/portal/resource/pt/biblio-883013> |
| Tozan *et al.* 2023 (47) | Impact, healthcare utilization and costs of travel-associated mosquito-borne diseases in international travelers: a prospective study | <https://pubmed.ncbi.nlm.nih.gov/37129519/> |
| Vázquez-Cruz *et al.* 2018 (48) | Costos e incapacidad por chikunguña en el Instituto Mexicano del Seguro Social em Guerrero, México | <https://www.redalyc.org/journal/4577/457754052012/html/> |
| Vazquez‐Prokopec *et al.* 2022 (54) | Preventive residual insecticide applications successfully controlled *Aedes aegypti* in Yucatan, Mexico | <https://pubmed.ncbi.nlm.nih.gov/36539478/> |
| Vidal *et al.* 2022 (57) | Epidemiological burden of Chikungunya fever in Brazil, 2016 and 2017 | <https://pubmed.ncbi.nlm.nih.gov/34905272/> |
| Vijayakumar *et al.* 2013 (49) | Economic impact of chikungunya epidemic: out-of-pocket health expenditures during the 2007 outbreak in Kerala, India | <https://pubmed.ncbi.nlm.nih.gov/23682438/> |
| **Cost-Outcome studies** | | |
| Claypool *et al.* 2019 (58) | Quantifying Positive Health Externalities of Disease Control Interventions: Modeling Chikungunya and Dengue | <https://pubmed.ncbi.nlm.nih.gov/31642362/> |
| Claypool *et al.* 2021 (59) | Prevention and control of dengue and chikungunya in Colombia: A cost-effectiveness analysis | <https://pubmed.ncbi.nlm.nih.gov/34965277/> |
| Guzzetta *et al.* 2017 (60) | Effectiveness and economic assessment of routine larviciding for prevention of chikungunya and dengue in temperate urban settings in Europe | <https://pubmed.ncbi.nlm.nih.gov/28892499/> |
| Trentini *et al.* 2018 (61) | The containment of potential outbreaks triggered by imported Chikungunya cases in Italy: a cost utility epidemiological assessment of vector control measures | <https://pubmed.ncbi.nlm.nih.gov/29899520/> |
| **Quality-of-life studies** | | |
| Barreto 2019 (62) | Impaired functionality and low quality of life of individuals affected by Chikungunya in Fortaleza-CE. 2019 | <https://repositorio.ufc.br/handle/riufc/48997?locale=es> |
| Couturier *et al.* 2012 (68) | Impaired quality of life after chikungunya virus infection: a 2-year follow-up study | <https://pubmed.ncbi.nlm.nih.gov/22427407/> |
| de Andrade *et al.* 2010 (63) | Chronic pain associated with the Chikungunya Fever: long lasting burden of an acute illness | <https://pubmed.ncbi.nlm.nih.gov/20170492/> |
| Doran *et al.* 2022 (69) | Long-term Chikungunya sequelae and quality of life 2.5 years post-acute disease in a prospective cohort in Curaçao | <https://journals.plos.org/plosntds/article?id=10.1371/journal.pntd.0010142> |
| Doran *et al.* 2022 (70) | The clinical manifestation and the influence of age and comorbidities on long‑term chikungunya disease and health‑related quality of life: a 60‑month prospective cohort study in Curaçao | <https://bmcinfectdis.biomedcentral.com/articles/10.1186/s12879-022-07922-1> |
| Elsinga *et al.* 2017 (71) | Long-term chikungunya sequelae in Curaçao: burden, determinants and a novel classification tool | <https://pubmed.ncbi.nlm.nih.gov/28931219/> |
| Hayd *et al.* 2020 (72) | Persistent chikungunya arthritis in Roraima, Brazil | <https://pubmed.ncbi.nlm.nih.gov/32170487/> |
| Jain *et al.* 2017 (73) | Clinical, Serological, and Virological Analysis of 572 Chikungunya Patients From 2010 to 2013 in India | <https://pubmed.ncbi.nlm.nih.gov/28379375/> |
| Marimoutou *et al.* 2015 (64) | Chikungunya infection: self-reported rheumatic morbidity and impaired quality of life persist 6 years later | <https://pubmed.ncbi.nlm.nih.gov/25752222/> |
| Panato *et al.* 2019 (65) | Evaluation of functional disability after Chikungunya infection | https://pubmed.ncbi.nlm.nih.gov/31778420/ |
| Paraense 2019 (66) | Assessment of quality of life in individuals diagnosed with Chikungunya fever | <https://bdtd.ibict.br/vufind/Record/IEC-2_85b6ae7c3b31f7daafdbd9d624a92d99> |
| Ramachandran *et al.* 2012 (67) | Impact of Chikungunya on Health-Related Quality of Life Chennai, South India | <https://www.ncbi.nlm.nih.gov/pmc/articles/PMC3520806/> |
| Simon *et al.* 2022 (74) | Determinants of Health-Related Quality of Life in Chronic Chikungunya Disease in Guadeloupe | <https://pubmed.ncbi.nlm.nih.gov/36145421/> |
| Watson *et al.* 2021 (75) | Tender and swollen joint counts are poorly associated with disability in chikungunya arthritis compared to rheumatoid arthritis | <https://pmc.ncbi.nlm.nih.gov/articles/PMC8448837/> |

Table 7S - Checklist to appraise the comprehensiveness, transparency and consistency of cost-of-illness (COI) studies*

| **PUBLICATIONS** | **DOMAIN** | | | | | | | | | | | | | | | | | | | | | | | | | | **QUALITY SCORE** |
| --- | --- | --- | --- | --- | --- | --- | --- | --- | --- | --- | --- | --- | --- | --- | --- | --- | --- | --- | --- | --- | --- | --- | --- | --- | --- | --- | --- |
|  | **INTRODUCTION** | | | | **METHODS AND COST ANALYSIS** | | | | | | | | | | | | | | | | **OUTCOMES AND REPORTS** | | | | | |  |
|  | **1** | **2** | **3** | | **4** | **5** | **6** | **7** | | **8** | | **9** | | **10** | | **11** | | **12** | | | **13** | **14** | **15** | **16** | | **17** |  |
|  |  |  | **A** | **B** |  |  |  | **A** | **B** | **A** | **B** | **A** | **B** | **A** | **B** | **A** | **B** | **A** | **B** | **C** |  |  |  | **A** | **B** |  |  |
| Alvis-Zakzuk *et al.* 2018 (33) | Y | Y | Y | Y | Y | Y | Y | Y | NA | Y | NA | Y | NA | Y | Y | NA | NA | Y | Y | P | Y | Y | Y | N | N | Y | High |
| Bloch 2016 (34) | Y | Y | Y | N | Y | Y | Y | Y | NA | Y | NA | Y | NA | Y | NA | NA | NA | Y | N | P | Y | Y | N | Y | N | N | Medium-high |
| Canali *et al.* 2017 (52) | Y | Y | Y | Y | Y | Y | Y | Y | NA | Y | NA | Y | NA | Y | N | N | NA | N | NA | NA | Y | Y | N | Y | Y | Y | High |
| Cardona-Ospina *et al.* 2015 (35) | Y | Y | Y | NA | Y | N | Y | Y | NA | Y | NA | Y | NA | Y | NA | NA | NA | Y | N | N | Y | Y | N | N | Y | Y | Medium-high |
| de Margarette et al 2022 (36) | Y | Y | Y | N | Y | Y | Y | Y | NA | Y | NA | Y | NA | Y | NA | NA | NA | Y | NA | NA | Y | Y | N | N | N | N | Medium-high |
| Feldstein *et al.* 2019 (37) | Y | Y | N | NA | Y | N | Y | Y | NA | Y | NA | Y | NA | N | NA | NA | NA | Y | N | NA | Y | Y | N | N | N | Y | Medium-high |
| Gonçalves 2021 (38) | Y | Y | Y | P | Y | Y | Y | P | NA | Y | NA | Y | NA | Y | Y | NA | NA | N | NA | N | Y | Y | N | N | N | N | Medium-high |
| Gopalan & Das 2009 (39) | Y | Y | Y | Y | Y | N | Y | Y | NA | Y | NA | Y | NA | Y | N | NA | NA | N | NA | N | Y | Y | N | Y | N | N | Medium-high |
| Heydari *et al.* 2017 (51) | Y | Y | Y | Y | Y | Y | Y | Y | NA | Y | NA | Y | NA | Y | N | NA | NA | N | NA | NA | Y | Y | Y | N | N | Y | Medium-high |
| Hossain *et al.* 2018 (40) | Y | Y | Y | Y | Y | Y | Y | Y | NA | Y | NA | Y | NA | Y | Y | NA | NA | N | NA | NA | Y | Y | Y | Y | N | N | High |
| Kaur *et al.* 2022 (50) | Y | Y | Y | Y | Y | Y | Y | Y | NA | Y | NA | Y | NA | N | NA | NA | NA | N | NA | NA | Y | Y | N | Y | Y | Y | High |
| Kolimenakis *et al.* 2019 (41) | Y | Y | Y | N | Y | Y | Y | Y | NA | Y | NA | Y | NA | Y | N | NA | NA | N | NA | NA | Y | Y | Y | N | N | Y | Medium-high |
| Krishnamoorthy *et al.* 2009 (42) | Y | Y | N | NA | Y | N | P | Y | NA | Y | NA | Y | NA | Y | NC | NA | NA | Y | N | P | Y | Y | Y | Y | N | Y | Medium-high |
| Nandha & Krishnamoorthy 2009 (43) | Y | Y | Y | Y | Y | P | Y | Y | NA | Y | NA | Y | NA | Y | Y | NA | NA | Y | N | Y | Y | N | Y | Y | Y | N | High |
| Salinas-López *et al.* 2018 (53) | Y | Y | Y | Y | Y | P | NA | Y | NA | Y | NA | Y | NA | Y | P | NA | NA | N | N | N | Y | Y | Y | N | N | N | Medium-high |
| Seyler *et al.* 2010 (44) | Y | Y | N | NA | Y | N | Y | Y | NA | Y | NA | Y | NA | N | NA | N | NA | Y | N | Y | Y | Y | N | N | Y | Y | Medium-high |
| Soumahoro *et al.* 2011 (45) | Y | Y | Y | Y | N | Y | Y | Y | NA | Y | NA | Y | NA | Y | N | NA | NA | N | NA | NA | Y | Y | Y | Y | Y | Y | High |
| Teich *et al.* 2017 (46) | Y | N | Y | Y | Y | Y | Y | Y | NA | Y | NA | Y | NA | Y | Y | NA | NA | Y | Y | Y | Y | P | Y | Y | Y | Y | High |
| Tozan *et al.* 2023 (47) | Y | Y | Y | N | Y | P | Y | Y | NA | Y | NA | Y | NA | N | NA | NA | NA | N | NA | NA | Y | Y | Y | N | N | N | Medium-high |
| Vázquez-Cruz et al 2018 (48) | Y | Y | Y | Y | Y | Y | Y | Y | NA | Y | NA | Y | NA | Y | Y | NA | NA | Y | Y | P | Y | Y | Y | P | P | Y | High |
| Vazquez‐Prokopec *et al.* 2022 (54) | Y | P | Y | Y | Y | N | Y | P | NA | P | NA | Y | NA | Y | NA | N | NA | N | NA | Y | Y | Y | Y | Y | N | N | Medium-high |
| Vijayakumar *et al.* 2013 (49) | Y | Y | Y | Y | Y | Y | Y | Y | NA | Y | NA | Y | NA | Y | Y | NA | NA | N | NA | NA | Y | Y | Y | Y | N | N | High |

Legend: Y = Yes; N = No; P = Partial; NA = Not applicable; NC = Not clear.

**Consensus-based checklist for cost-of-illness studies*

| **Study characteristics** | | |
| --- | --- | --- |
| Question/objective | 1) | Is a well-defined research question or objective stated? |
| Population | 2) | Is the study population described? |
| Perspective | 3) | a) Is (are) the chosen study perspective(s) stated? |
|  |  | b) If so, is (are) the chosen study perspective(s) justified? |
| **Methodology and cost analysis** | | |
| Epidemiological approach | 4) | Is the epidemiological approach reported (e.g., prevalence, incidence)? |
| Costing approach | 5) | Is the costing approach reported (e.g., top-down, bottom-up)? |
| Data collection approach | 6) | Is the data collection process reported (e.g., prospective, retrospective)? |
| Identification | 7) | a) Are all components of resource use identified that are relevant to the condition/disease, population, intervention, study objectives, and study perspective? |
|  |  | b) If not, is a justification provided for excluding relevant components of resource use? |
| Measurement | 8) | a) Are all included components of resource use measured? |
|  |  | b) If not, is a justification provided for not measuring certain components of resource use? |
| Valuation | 9) | a) Are all included components of resource use valued in monetary terms? |
|  |  | b) If not, is a justification provided for not valuing certain components of resource use? |
| Time horizon | 10) | a) Is the chosen time horizon specified? |
|  |  | b) If so, is the chosen time horizon justified? |
| Discounting | 11) | a) Are future costs discounted? |
|  |  | b) If so, is a justification provided for the discount rate? |
| Sensitivity | 12) | a) Are all variables whose values are uncertain subjected to sensitivity analysis? |
|  |  | b) If so, is a justification provided for which variables are subjected to sensitivity analysis? |
|  |  | c) Are analyses done on relevant subgroups? |
| **Results and reporting** | | |
| Cost sectors | 13) | Are the study results presented transparently by cost category/sector? |
| Generalizability | 14) | Do the authors discuss the generalizability of study results (e.g., comparing the results to other patient/client groups or/in other settings)? |
| Limitations | 15) | Do the authors discuss important limitations? |
| Ethical and distributional issues | 16) | a) Do the authors discuss ethical issues? |
|  |  | b) Do the authors discuss distributional issues? |
| Conflict of interest | 17) | Do the authors report any potential conflicts of interest? |

**Source:** Schnitzler L, Roberts TE, Jackson LJ, Paulus ATG, Evers SMAA. A consensus-based checklist for the critical appraisal of cost-of-illness (COI) studies. International Journal of Technology Assessment in Health Care. 2023;39(1):e34. doi:10.1017/S0266462323000193

**Table 8S** - Checklist for critical analysis of published articles on economic evaluation of health technologies

| **Checklist** | **Publications** | | | |
| --- | --- | --- | --- | --- |
|  | Claypool *et al.* 2021 (59) | Claypool *et al.* 2019 (58) | Guzzetta *et al.* 2017 (60) | Trentini *et al.* 2018 (61) |
| **a) Study Design** | | | | |
| Was the study question clearly defined, appropriately framed, and answerable? | Y | Y | Y | Y |
| Was the target population clearly described? | Y | Y | Y | Y |
| Were the main alternatives included and comprehensively described? | Y | Y | Y | Y |
| Was the model's time horizon long enough to capture meaningful differences in costs and health outcomes between the strategies analyzed? | Y | Y | NC | Y |
| Was the study perspective stated? | Y | N | Y | Y |
| Did the study assess both costs and health outcomes? | Y | Y | Y | Y |
| Was the type of economic evaluation specified? | Y | Y | Y | Y |
| **b) Measurement of Health Outcomes and Costs Health Outcomes** | | | | |
| 8. Were the health outcome measures clearly described and appropriate to the study question? | Y | Y | Y | Y |
| 9. Were the sources of health outcome estimates described, justified, and aligned with the target population? | Y | Y | Y | Y |
| 10. Were methods and assumptions used to extrapolate short-term outcomes into medium- or long-term outcomes described and justified? | NA | NA | NA | NA |
| 11. Did the research protocol reflect typical clinical practice if outcome estimates were derived from clinical trials? | NA | NA | NA | NA |
| 12. If outcome estimates were based on systematic reviews, was the quality of the evidence reported? | NA | NA | NA | NA |
| 13. If outcome estimates came from observational studies or assumptions, was their use due to the absence of higher-quality evidence? | NC | NC | NC | NC |
| ***Costs*** | | | | |
| 14. Were costs clearly described? | Y | Y | Y | Y |
| 15. Was cost measurement consistent with the study perspective? | Y | NC | Y | Y |
| 16. Was the cost estimation method described and appropriate? | Y | Y | Y | Y |
| 17. Was information provided regarding the currency and the period during which the costs were collected? | Y | Y | N | N |
| 18. Was inflation adjustment applied if costs were collected over different periods? | Y | Y | N | N |
| 19. Were future costs and outcomes discounted using the same and appropriate discount rate? | Y | Y | N | Y |
| **c) Analysis and Interpretation of Results** | | | | |
| ***Analytical Model*** | | | | |
| 20. Was an analytical model used, and was it appropriate for the study objectives? | Y | Y | Y | Y |
| 21. Did the health states represented in the model reflect the biological course of the disease and the consequences of the technologies under evaluation? | Y | Y | Y | Y |
| 22. Was methodological uncertainty addressed? | Y | NC | Y | NC |
| 23. Was structural uncertainty addressed? | Y | Y | Y | Y |
| 24. Was heterogeneity addressed? | NA | Y | NA | NA |
| 25. Was parameter uncertainty addressed? | Y | Y | Y | Y |
| ***Results*** | | | | |
| 26. Were the study results presented using a cost-effectiveness or cost-utility ratio? | Y | Y | Y | Y |
| 27. Was the discussion of results sufficiently broad, including key aspects relevant to patients and decision-makers? | Y | Y | Y | Y |
| 28. Was internal model consistency reported? | Y | N | N | Y |
| 29. Was external model consistency reported? | N | N | Y | Y |
| **General Information** | | | | |
| 30. Was the study’s source of funding appropriately disclosed? | Y | Y | Y | Y |
| 31. Did the authors declare potential conflicts of interest? | Y | N | Y | Y |
| 32. Did an institutional review board or ethics committee approve the study? | N | N | N | N |
| **QUALITY SCORE** | **High** | **Medium- high** | **Medium- high** | **High** |

**Source:** Silva EN, Galvão TF, Pereira MG, Silva MT. Estudos de avaliação econômica de tecnologias em saúde: roteiro para análise crítica. Rev Panam Salud Publica. 2014;35(3):219–27.

**Table 9S –** Checklist for analytical cross-sectional studies

| **Publications** | **JBI Critical Appraisal Checklist for Analytical Cross-Sectional Studies** | | | | | | | | **QUALITY SCORE** |
| --- | --- | --- | --- | --- | --- | --- | --- | --- | --- |
|  | **1. Were the criteria for inclusion in the sample clearly defined?** | **2. Were the study subjects and the setting described in detail?** | **3. Was the exposure measured in a valid and reliable way?** | **4. Were objective, standard criteria used for measurement of the condition?** | **5. Were confounding factors identified?** | **6. Were strategies to deal with confounding factors stated?** | **7. Were the outcomes measured in a valid and reliable way?** | **8. Was appropriate statistical analysis used?** |  |
| Barreto 2019 (62) | Y | Y | Y | Y | NA | NA | Y | Y | High |
| Couturier *et al.* 2012 (68) | Y | Y | Y | Y | Y | Y | Y | Y | High |
| de Andrade *et al.* 2010 (63) | Y | Y | Y | Y | Y | Y | Y | Y | High |
| Doran *et al.* 2022 (69) | Y | Y | Y | Y | Y | Y | Y | Y | High |
| Doran *et al.* 2022 (70) | Y | Y | Y | Y | Y | Y | Y | Y | High |
| Elsinga *et al.* 2017 (71) | Y | Y | Y | Y | N | NA | Y | Y | High |
| Hayd *et al.* 2020 (72) | Y | Y | Y | Y | Y | Y | Y | Y | High |
| Jain *et al.* 2017 (73) | Y | Y | Y | Y | Y | Y | Y | Y | High |
| Man *et al.* 2022 (55) | Y | Y | Y | Y | NA | NA | Y | Y | High |
| Marimoutou *et al.* 2015 (64) | Y | Y | Y | Y | N | NA | Y | Y | High |
| Mora-Salamanca *et al.* 2020 (56) | Y | Y | Y | Y | Y | Y | Y | Y | High |
| Panato *et al.* 2019 (65) | Y | Y | Y | Y | N | NA | Y | Y | High |
| Paraense 2019 (66) | Y | Y | Y | Y | Y | Y | Y | Y | High |
| Simon *et al.* 2022 (74) | Y | Y | Y | Y | N | NA | Y | Y | High |
| Ramachandran *et al.* 2012 (67) | Y | Y | Y | Y | Y | Y | Y | Y | High |
| Vidal *et al.* 2022 (57) | Y | Y | Y | Y | Y | Y | Y | Y | High |
| Watson *et al.* 2021 (75) | Y | Y | Y | Y | Y | Y | Y | Y | High |

Legend: Y = Yes; N = No; P = Partial; NA = Not applicable; NC = Not clear.

**Source:** Moola S, Munn Z, Tufanaru C, Aromataris E, Sears K, Sfetcu R, Currie M, Qureshi R, Mattis P, Lisy K, Mu P-F. Chapter 7: Systematic reviews of etiology and risk. In: Aromataris E, Munn Z (Editors). Joanna Briggs Institute Reviewer's Manual. The Joanna Briggs Institute, 2017. Available from <https://reviewersmanual.joannabriggs.org/>
